# Coumarin‐Caged Nanoparticle for Light‐Driven Surface Modification

**DOI:** 10.1002/cmdc.202500636

**Published:** 2025-10-07

**Authors:** Jan Birringer, Johannes Konrad, Stephan Melchner, Marius Remmert, Achim Goepferich

**Affiliations:** ^1^ Department of Pharmaceutical Technology University of Regensburg Regensburg 93053 Bavaria Germany

**Keywords:** cell‐penetrating peptides, charge‐mediated uptake, nanoparticles, stimuli‐responsive, surface chemistry

## Abstract

Photo‐labile protecting groups (PPG) allow for the selective activation of an originally caged moiety by light exposure at a specific wavelength. Incorporation of PPG in nanoparticles (NPs) enables precise spatiotemporal control over NPs surface properties. Thus, physicochemical and biological properties of NPs can be modified even after administration in a biological environment. In this study, this mechanism is used to control the cell uptake of NPs. To this end, polymeric core–shell NPs are used composed of poly(D, L‐lactide‐co‐glycolide) and a poly(ethylene glycol)‐b‐poly(D, L‐lactide) block copolymer, modified with positively charged cell‐penetrating peptide (CPP). Surface charge of CPP‐NPs (+23.50 mV), measured as zetapotential, is effectively diminished by the attachment of coumarin‐derived PPG to CPP (+12.50 mV), resulting in reduced cell uptake. Upon light irradiation with light‐emitting diode (*λ *= 365 nm) the PPG is cleaved, restoring the zetapotential (+24.67 mV) and triggering an enhanced cell uptake. This opens the door to trigger the cellular uptake of NPs that are intended to transport drugs to their target cells in the future.

## Graphical abstract

Photo‐responsive nanoparticles (NPs) enable spatiotemporal control over drug delivery. Attachment of a photo‐labile protecting group (PPG) to cell‐penetrating peptide (CPP) effectively camouflages the positive charge of NPs. Light irradiation cleaves the PPG, exposing the CPP on the NP surface and thereby enhancing cellular uptake.

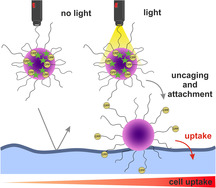

## 1 Introduction

Polymeric nanoparticles (NPs) are regarded as a promising platform for targeted drug delivery and controlled drug release to cells. However, despite active targeting concepts, NPs reach the targeted tissue only in a single‐digit percentage range. Consequently, more than 90% of the applied NPs can cause undesired effects at off‐target sites.^[^ ^1^ ^]^ To achieve enhanced control over the NPs’ biodistribution and to increase their tissue selectivity, the design of modular physicochemical properties has recently attracted more attention.^[^ ^2^ ^,^ ^3^ ^]^ Especially, modification of properties after NP administration in a biological environment are of high interest as these dynamic surface modifications mirror adaptive strategies employed by viruses.^[^ ^4^ ^,^ ^5^ ^]^ To obtain adaptable NPs, moieties responding to either internal (pH, enzymes, redox) or external (light, magnetism, temperature) stimuli can be implemented.^[^ ^6^ ^,^ ^7^ ^]^ Light is an excellent external trigger as it is non‐invasive and offers high spatial and temporal resolution, and control.^[^ ^8^ ^]^ However, light penetration in tissues remains challenging. Especially in the visible and ultraviolet region, tissue scatters, and significantly absorbs light. Thus, light suffers from a rather limited penetration depth and is only reasonably applicable to tissues that can be directly penetrated, such as the vitreous of the eyes.^[^ ^9^, ^10^, ^11^ ^–^ ^12^ ^]^ Despite this limitation, innovative strategies to use light for photo‐controlled drug release or drug delivery have been developed as it is independent of the biological environment.^[^ ^13^, ^14^ ^–^ ^15^ ^]^

**Figure 1:**
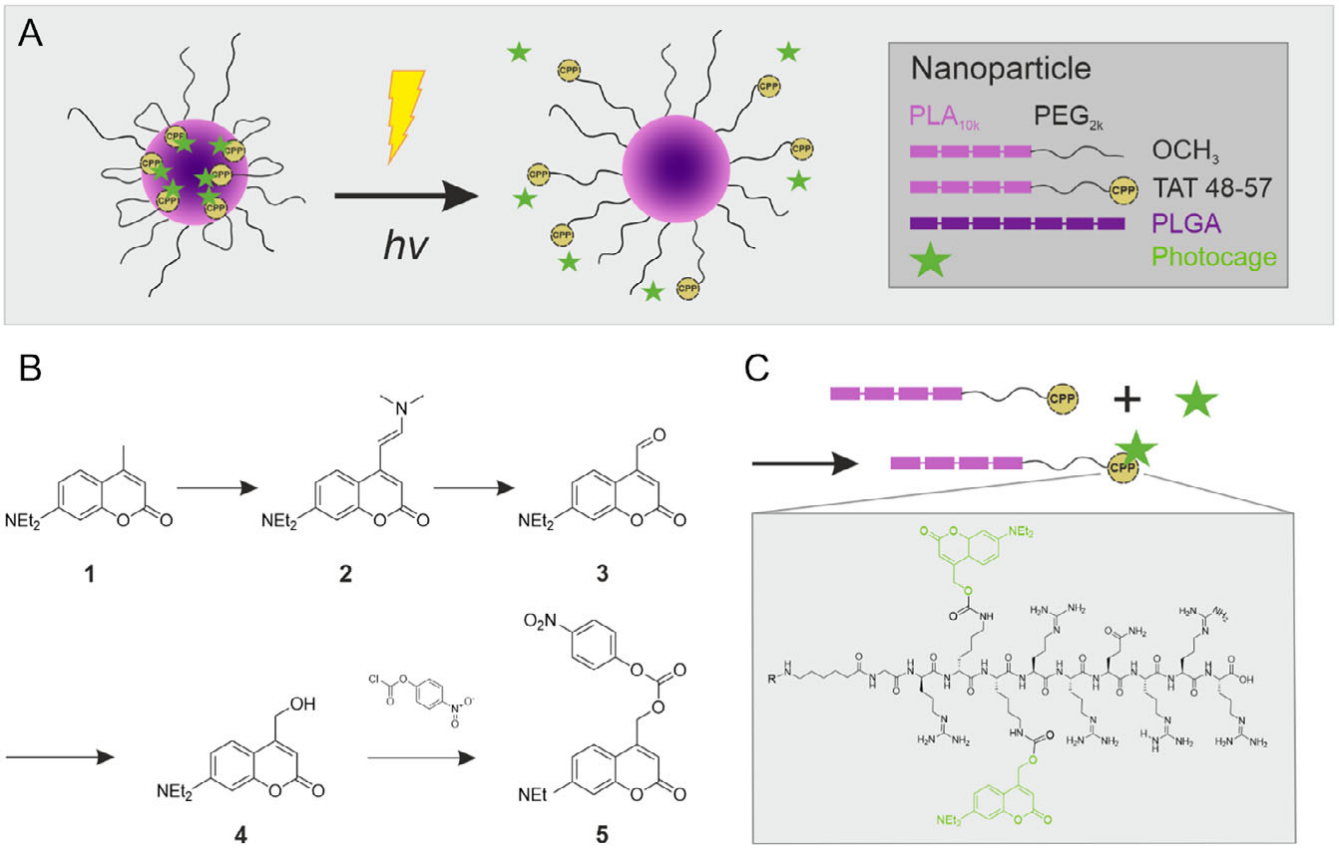
Schematic representation of photo‐responsive NP and synthesis of PPG. A) Photo‐responsive core–shell NP composed of PLGA and a PLA‐PEG block copolymer, with its surface functionalized by photocaged CPP. B) Synthesis scheme of coumarin‐derived PPG. C) Schematic synthesis of photocaged TAT‐polymer. The amino acid sequence of TAT 48–57 (CPP) is depicted in the gray box. Coumarin‐derived PGG (green) is attached to the lysine residues of TAT 48–57.

In terms of photo‐controlled drug delivery, photo‐labile protecting groups (PPG), also referred to as “photocage”, are commonly used. PPG can, thereby, mask the biological function of a moiety, such as cell‐penetrating peptides (CPP), providing spatiotemporal control over their visibility.^[^ ^16^ ^]^ CPPs have the ability to penetrate cell membranes and facilitate the intracellular delivery of associated cargos.^[^ ^17^ ^,^ ^18^ ^]^ Furthermore, it is hypothesized that CPPs promote endosomal escape.^[^ ^19^, ^20^ ^–^ ^21^ ^]^ Consequently, CPPs are considered a precious tool for NP surface functionalization, improving intracellular drug delivery. The transactivator of transcription (TAT) protein from human immunodeficiency virus, one of the most studied CPPs, is rich in basic amino acids (2 lysine and 6 arginine), causing a highly positive net charge.^[^ ^22^ ^]^ Functionalizing NPs with TAT alters their surface charge and increases NP uptake.^[^ ^23^ ^]^ The incorporation of coumarin‐derived PPG (DEAC) into the TAT sequence masks some of the positive charges that mediate cell uptake and cause the hiding of the sequence inside the NP corona (**Figure** **1**A,C).^[^ ^12^ ^,^ ^24^ ^]^ Upon light irradiation the PPG is cleaved, presenting TAT on the NP surface restoring the CPP's penetration enhancing properties.

In the work of Wang et al., this caging concept was already established using polymeric micelles consisting of poly(ethylene glycol)‐b‐poly(D, L‐lactide) (PEG‐PLA) block copolymer, which are relatively stable in vitro.^[^ ^12^ ^,^ ^25^ ^]^ However, in vivo stability of block copolymer micelles is poor with a dissociation time of less than 10 min.^[^ ^26^ ^]^ However, long circulation times of NP in blood are crucial for their use in chronic disease treatment as otherwise repeated dosing in brief intervals is necessary. In contrast to polymeric micelles, NPs have better stability in vivo. Core–shell NPs consisting of poly(D, L‐lactide‐co‐glycolide) (PLGA) and PEG‐PLA block copolymer for example are stable for at least 10 days.^[^ ^27^ ^]^

Stability of drug delivery system is intrinsically linked with the release of the incorporated drug. Thus, poor stability of micelles can lead to premature drug release causing undesirable side effects or even worse, decreasing the therapeutic efficacy.^[^ ^26^ ^,^ ^28^ ^,^ ^29^ ^]^ Reports in the literature have demonstrated that NPs composed of a core material with high molecular weight retain hydrophobic drugs better than micelles and have an increased circulation time in the blood.^[^ ^30^ ^]^

In addition to enhanced stability, NPs offer significant advantages in ligand presentation and molecular engineering. Like liposomes, which are known for their membrane fluidity, copolymeric micelles exhibit characteristic single‐chain exchange.^[^ ^31^ ^,^ ^32^ ^]^ Dynamic rearrangement of polymer chains might cause functional loss of hydrophobic ligands as these moieties can become embedded in micellar core.^[^ ^33^ ^]^ In core–shell NP design, the mobility of block copolymer is reduced due to their anchoring within the hydrophobic core. However, this assembly preserves flexibility allowing ligand mobility and enhanced avidity.^[^ ^34^ ^]^ Moreover, the use of solid core NPs facilitate stable surface engineering.^[^ ^35^ ^]^

Therefore, our goal was to scrutinize whether the concept of camouflaging CPPs with PPGs could also be used for NPs. The specific aim of this study was to design a NP system, that offers spatiotemporal control over NP uptake by target cells.

## 2 Results and Discussion

### 2.1 Synthesis and Characterization of Photo‐Responsive NPs

For investigation of light‐controlled uptake of NPs into cell, the components of core–shell NP were modified. To study uptake behavior in vitro, fluorescence dye Cyanine5 (Cy5) was tethered to PLGA, enabling NP visualization in flow cytometry and confocal microscopy. PLA‐PEG was functionalized with CPP TAT protected by a coumarin‐derived PPG [TAT] and TAT without any modification. Therefore, TAT was synthesized with a purity of 89% and coupled to COOH‐PEG_2k_‐PLA_10k_ with a coupling efficiency (CE) of greater than 90%. The coumarin‐derived PPG (7‐(diethylamino)‐4‐(hydroxymethyl)‐coumarin, DEAC) was synthesized with a purity of greater than 90% and subsequently coupled to TAT‐PEG_2k_‐PLA_10k_ with an efficiency of 56% ([TAT]‐PEG_2k_‐PLA_10k_). In a previous study, it was shown that TAT‐NP with a degree of 50% functionalization (DOF) resulted in a positive surface charge, measured as particle zetapotential.^[^ ^23^ ^]^ Therefore, the same DOF for TAT‐NP, as well as [TAT]‐NP, was used in the present study.

**Figure 2:**
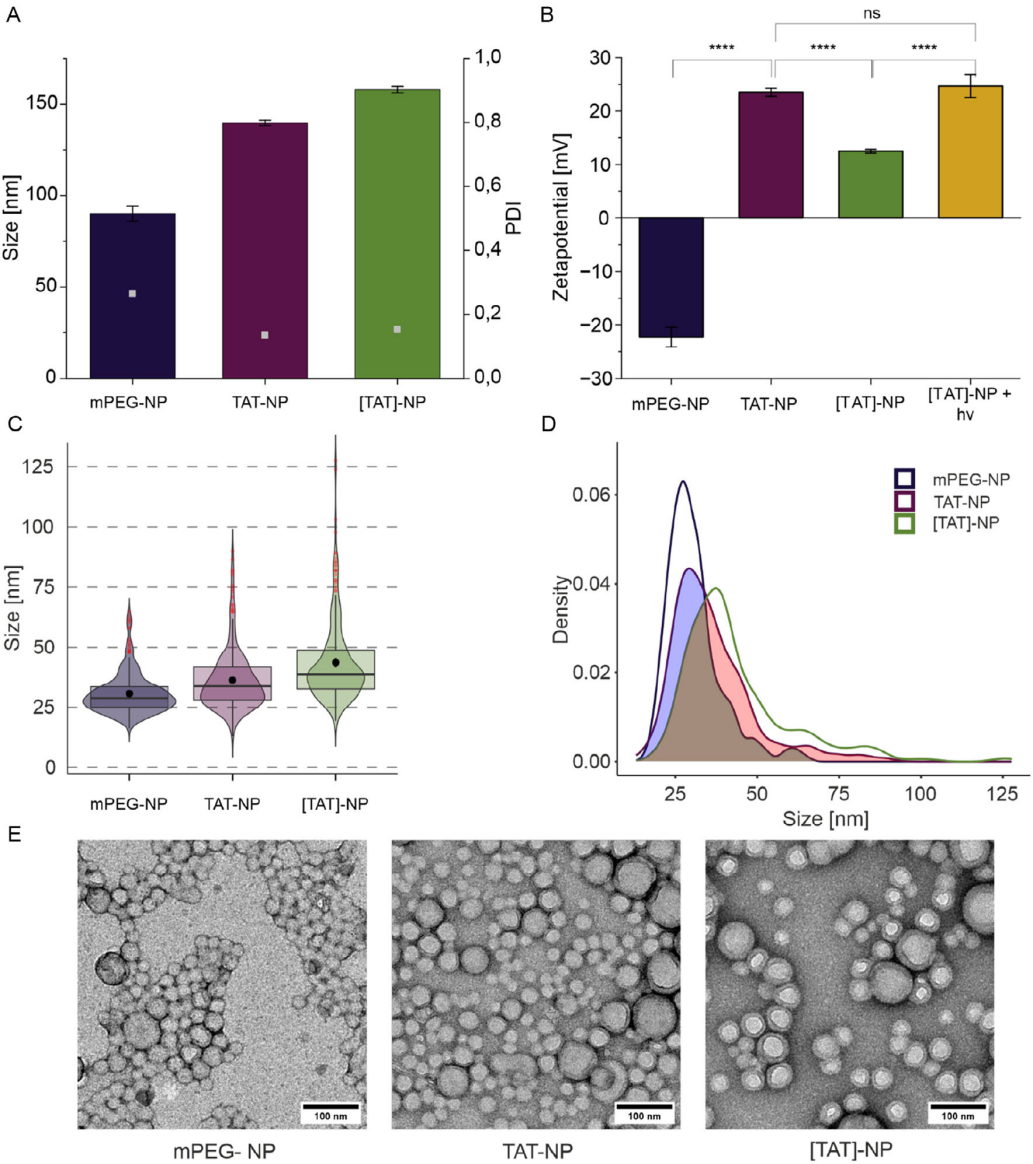
Characterization of NPs. A) Size as hydrodynamic diameter (*d*_h_) and PDI. B) Zetapotentials. Data represent mean ± SD (*n *= 3, level of statistical significance is indicated as * *p *≤ 0.05, ** *p *≤ 0.01, *** *p *≤ 0.001, **** *p *≤ 0.0001). C) Violin plot represents size distribution of diameters measured from TEM images. The median NP size is shown as horizontal line within the box, whose lower and upper edges represent the first (Q1) and third (Q3) quartiles, respectively. Whiskers indicate the most extreme data points within 1.5 times the interquartile range from the lower and upper quartiles. Points beyond this range are shown individually as outliers and are displayed as individual points in red. 95% confidence interval for the mean is represent by black dot with bars. Mean size of NPs differed significantly (Welch‐Test, see Figure S18, Supporting Information). A total of 300 NPs were measured. D) Density curve of NP size distribution and overlap. The density curves illustrate the smoothed distribution of sizes for each NP. Overlap between size distributions of NP types are indicated as shaded areas. A total of 300 NPs were measured. E) Representative TEM micrographs of each NP formulation. Scale bars are indicated in each panel.

Methoxy‐terminated polymer chain‐derived NP (mPEG‐NP) served as control. All NPs were characterized in terms of size, size distribution, and surface charge (**Figure** **2**). The control NP were smaller in size (90.2 ± 4.16 nm) than TAT‐functionalized NP (139.73 ± 1.36 nm) and photocaged TAT‐NP (157.93 ± 1.74 nm) (Figure 2A). However, the functionalized NPs showed higher conformity as reflected by lower polydispersity index (PDI) value (mPEG‐NP: PDI = 0.266; TAT‐NP: PDI = 0.136; [TAT]‐NP: PDI = 0.153; Figure 2A). The larger size of functionalized NPs can be explained by their composition. TAT is rich in positively charged amino acids, which are extensively solvated, and thus resulting in larger hydrodynamic diameter (*d*_h_). Size increase of [TAT]‐NP in comparison to TAT‐NP can be explained by the orientation of DEAC toward the NP core, which results in combination with the higher molecular mass to a larger size.

Huge difference in *d*_h_ of functionalized NPs in comparison to control NP raised the question of comparability in uptake experiments. Therefore, the actual size of core–shell NPs was determined via transmission electron microscopy (TEM). The violin plot (Figure 2C) depicts the unimodal distribution of NP sizes within the analyzed sample, highlighting both the median and mean values, as well as the variability in size. Comparison of the mean sizes revealed that mPEG NP still exhibited the smallest in diameter (30.67 ± 8.36 nm), followed by TAT‐NP (36.16 ± 12.62 nm), while [TAT]‐NP displayed the largest size of 43.52 ± 16.57 nm. In Figure 2D, the density plot of size distribution of studied NPs is shown. The shaded regions indicate areas of overlap between NP types, highlighting similarities in their size distributions. The overlap value (value: 1 = complete overlap) was calculated for each NP pairing. The ratios (mPEG‐NP—TAT‐NP: 0.766; mPEG‐NP—[TAT]‐NP: 0.555; TAT‐NP—[TAT]‐NP: 0.782) indicate that NPs are more alike than NP tracking analysis (NTA) measurements and mean size values suggest. Functionalized NPs exceeded the desired size of <100 nm for in vivo experiments. However, for this proof of concept study we were not concerned and for future experiments, the size of functionalized NPs could be reduced by using a microfluidic synthesis that would provide us with smaller and uniformer NPs.^[^ ^36^ ^]^

As the charge of CPPs plays a crucial role in their ability to facilitate cellular uptake, the zetapotential of NPs was of high interest. Zetapotentials of mPEG and functionalized NPs are depicted in Figure 2B. Control NP had a negative zetapotential of −22.27 mV. As expected, functionalization of NP led to a change in surface charge. TAT‐NP had a positive surface charge of +23.50 mV. By incorporation of DEAC into the TAT sequence we aimed to mask the NP's positive charge. The positive charge was partially masked and the photocaged NP had a diminished positive zetapotential of +12.50 mV. Irradiation of [TAT]‐NP with light for 1 min ([TAT]‐NP + hv 1 min) led to a positive surface charge of +24.67 mV. The zetapotential reduction of [TAT]‐NP to approximately half of the zetapotential value of TAT‐NP, can be explained by incomplete linkage of DEAC to TAT. As CE of DEAC to TAT‐PEG_2k_‐PLA_10k_ was 56%, [TAT]‐NP actually consisted of [TAT]‐PEG_2k_‐PLA_10k_ as well as TAT‐PEG_2k_‐PLA_10k_ polymer.

### 2.2 Photocleavage of DEAC

**Figure 3:**
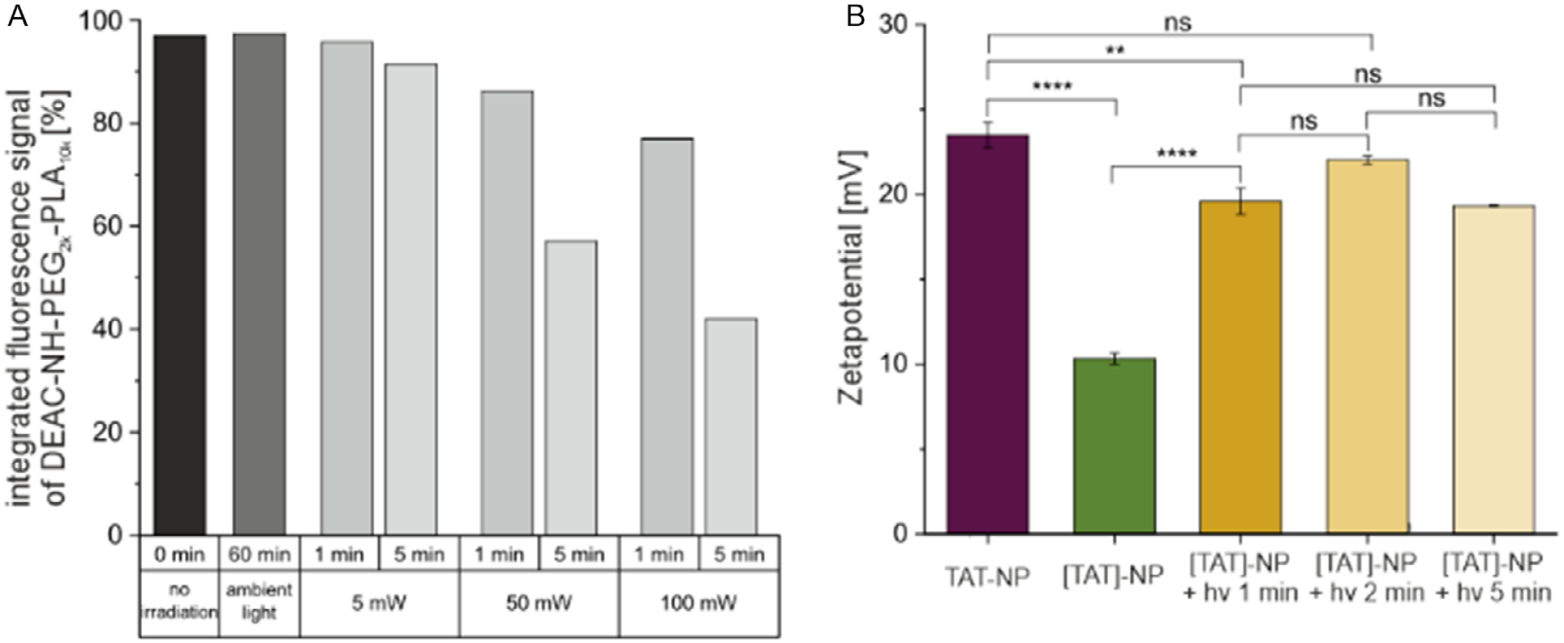
Photocleavage of DEAC from polymer. A) The integral of fluorescence detector (FLD) signal *λ*_ex _= 375 nm, *λ*_em _= 480 nm at the retention time of DEAC‐NH‐PEG_2k_‐PLA_10k_ as a function of different irradiation times and LED powers. Irradiation experiments were performed using a LED with an emission wavelength *λ*_em _= 365 nm. B) Zetapotential of TAT‐NP and [TAT]‐NP without and with LED irradiation. For irradiated samples no significant difference in zetapotential were measured. The LED power was set to 100 mW, *λ*_em _= 365 nm. Data represent mean ± SD (*n *= 3, level of statistical significance is indicated as * *p *≤ 0.05, ** *p *≤ 0.01, *** *p *≤ 0.001, **** *p *≤ 0.0001).

To confirm sufficient uncaging potential and gain information about the stability of DEAC caged polymer, uncaging experiments with a model polymer were conducted. DEAC was coupled via an amide bond to amino‐terminated PEG‐PLA (DEAC‐NH‐PEG_2k_‐PLA_10k_) representing the linkage of DEAC to *ε*‐amino group of lysine in the [TAT]‐polymer conjugate. Photocleavage of DEAC from the polymer led to a decrease in the integrated fluorescence signal of DEAC‐NH‐PEG_2k_‐PLA_10k_. We found that the DEAC modified model polymer is stable for at least 1 h under ambient light as no change in signal intensity was detected (**Figure** **3**A). This is important as all experiments, especially cell culture experiments, would not to be performed under light exclusion. The longer the irradiation time, respectively, the higher the light‐emitting diode (LED) power, the higher was the uncaging yield (Figure 3A).

More important than the investigation of the uncaging efficiency of the model polymer under light irradiation, was the reconstitution of characteristics of TAT‐NP. Therefore, uncaging experiments with zetapotential readout were performed. TAT‐NP had a zetapotential of +24.67 mV that dropped after the incorporation of DEAC into the TAT sequence to +10.32 mV. Irradiation with 100 mW for 1 min was sufficient to uncage the NPs since their zetapotential (+19.60 mV) was in the same range as that of the TAT‐NP (Figure 3B). Increasing irradiation time to 2 min resulted in a greater zetapotential (+22.03 mV), but the differences in zetapotential for all irradiated samples were not statistically significant. Therefore, the irradiation time was set to 1 min for the cell experiments. Performing uncaging experiments with [TAT]‐NP resulted in a loss of color of respective aqueous NP solutions (Figure S17B, Supporting Information). Consequently, photobleaching of Cy5 incorporated into the NPs was investigated.

### 2.3 Spectral Properties of DEAC and Conjugates

**Figure 4:**
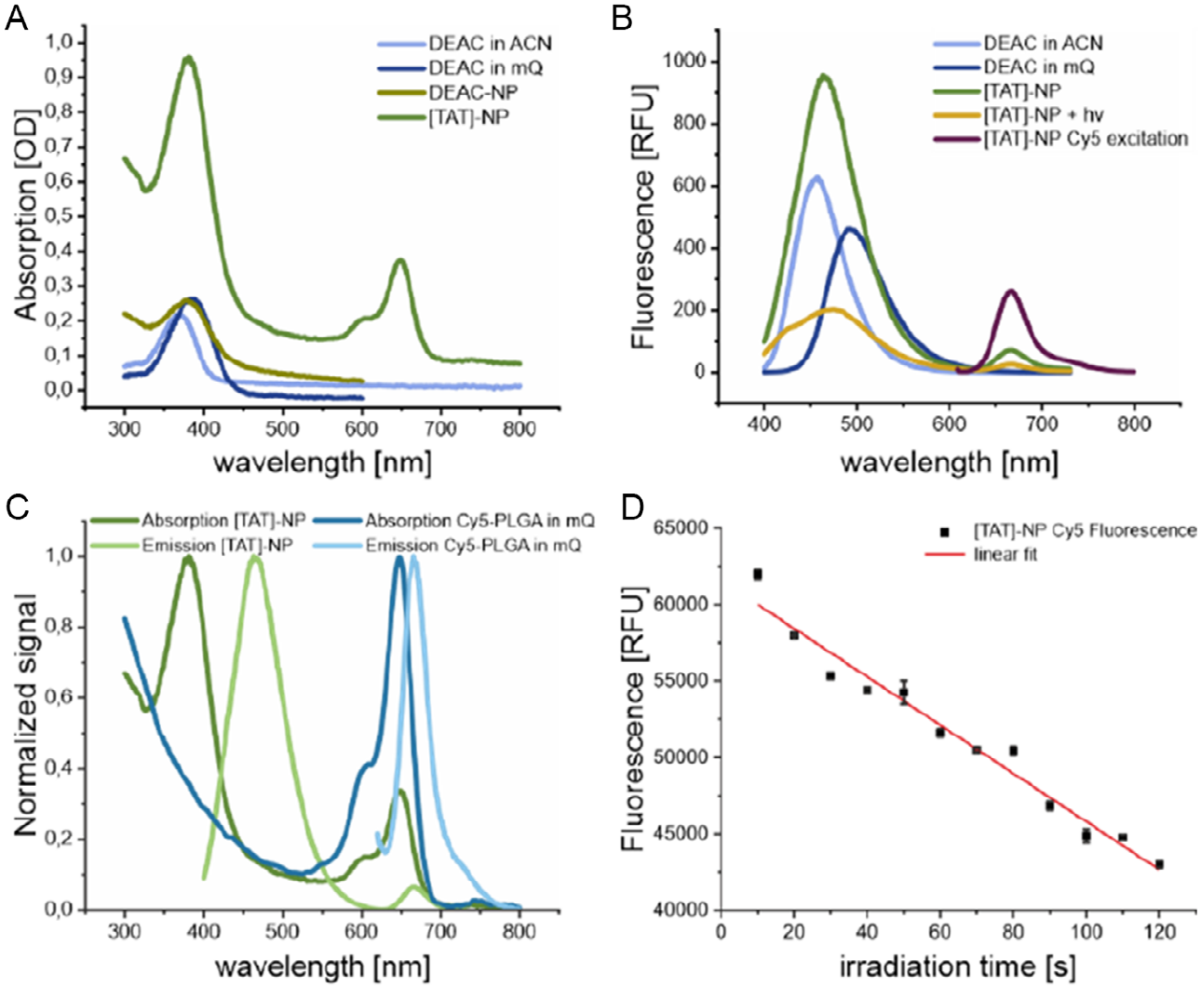
Spectral properties of DEAC and photo‐responsive NP. A) Absorption spectrum of DEAC in ACN (*λ*_max _= 368 nm), DEAC in Millipore water (mQ) (*λ*_max _= 384 nm), DEAC‐NP (made of DEAC‐NH‐PEG_2k_‐PLA_10k_) (*λ*_max _= 377 nm), and [TAT]‐NP (*λ*_max _= 381 nm, *λ*_max _= 648 nm). The first peak (*λ *≈ 380 nm) is related to DEAC, whereas second peak (*λ*_max _= 648 nm) is caused by Cy5 in the NP core. B) Fluorescence emission spectra of DEAC in ACN (*λ*_em,max _= 457 nm) and mQ (*λ*_em,max _= 493 nm), [TAT]‐NP (*λ*_em,max _= 463 nm), and [TAT]‐NP irradiated for 1 min (*λ*_em,max _= 474 nm) at an excitation wavelength of *λ*_ex _= 380 nm. Upon excitation at *λ*_ex _= 600 nm, Cy5 exhibited a fluorescence emission spectrum with a maximum at *λ*_em,max _= 668 nm (referred to as [TAT]‐NP Cy5 excitation). C) Absorption and fluorescence emission spectra of [TAT]‐NP and Cy5‐PLGA. Individual peaks were normalized to their highest peak intensity to enable comparison between spectra. [TAT]‐NP *λ*_abs,max1 _= 381 nm, *λ*_abs,_ _max2_ = 648 nm, *λ*_em,max1 _= 463 nm, *λ*_em,max2 _= 668 nm. Cy5‐PLGA *λ*_abs,max _= 647 nm, *λ*_em,max _= 666 nm. D) Photobleaching of Cy5 after LED irradiation of [TAT]‐ NP at *λ *= 365 nm; LED power: 100 mW. Fluorescence of Cy5 (RFU, relative fluorescence units) is plotted against irradiation time of NP.

Besides quantification of reaction yields, photophysical properties of synthesized photocage DEAC, caged polymer and fluorescent NPs were measured to characterize our NP design. We hypothesized that the hydrophobicity of DEAC would cause an orientation of [TAT] toward the hydrophobic PLGA core of [TAT]‐NP and thus, mask to some extent the positive charge of TAT. This hypothesis was based on two previous studies.^[^ ^12^ ^,^ ^24^ ^]^ To support our hypothesis, spectral measurements were performed. The absorption spectra of DEAC and conjugates in acetonitrile (ACN) and aqueous solution (**Figure** **4**A) are similar in shape and differ only slightly in their maxima due to solvent polarity and different linkage. In comparison to absorption, the polarity of the solvent has a strong impact on the fluorescence spectrum of the coumarin‐derived PPG.^[^ ^37^ ^]^ Thus, fluorescence was used to characterize the core–shell NPs. In ACN, a hydrophobic surrounding, the fluorescence spectrum of DEAC was found to be blueshifted (*λ*_em _= 457 nm) in comparison to an aqueous solution (*λ*_em _= 493 nm) (Figure 4B). Since the fluorescence spectrum of [TAT]‐NP (*λ*_em _= 463 nm) resembled that to DEAC in ACN and was blueshifted relative to DEAC in aqueous solution, we concluded that DEAC is located in a hydrophobic environment. This suggests that DEAC is orientated toward the core. After irradiation of [TAT]‐NP with light at *λ*_em _= 365 nm for 1 min, the fluorescence spectrum of DEAC was redshifted. The emission shift indicates a structural change of NP and that DEAC is in a more hydrophilic environment (Figure 4B,C). The lower fluorescence signal can be explained by photobleaching. Excitation of [TAT]‐NP at *λ*_ex _= 380 nm led to an additional emission peak with *λ*_em _ = 668 nm. This spectrum belongs to the fluorescent dye, Cy5, incorporated in the PLGA core, which is normally not excited at that wavelength (Figure 4B). The energy for Cy5 excitation is provided by Foerster resonance energy transfer (FRET), proofing proximity of DEAC and Cy5. Calculation of the Foerster radius (R_0_) of this pair of dyes resulted in 3.26 nm (see Supporting Information, Equation 12). At the distance of R_0_ the energy transfer efficiency between a donor and acceptor is 50%. At longer distances, the efficiency declines at a rate proportional to the sixth power, thereby reducing the probability of FRET.^[^ ^38^ ^]^ The PEG layer of core–shell NP had a thickness of 8.28 nm (see Supporting Information, Equation 17). Consequently, the distance between DEAC and Cy5 would be greater than twice R_0_ if DEAC would be located on the surface of the NP, rendering FRET highly unlikely. Thus, the appearance of FRET supports the hypothesis that DEAC anchored TAT within the NP. As the extent of NP uptake into HeLa cells is quantified by fluorescence readout of Cy5, photobleaching of Cy5 could have a tremendous impact on the outcome of flow cytometry experiments. Therefore, a bleaching factor *κ* was determined to take the reduced fluorescence of irradiated sample into account. In Figure 4D, the fluorescence signal of Cy5 is plotted against different irradiation times. [TAT]‐NPs shown a strong decrease in fluorescence signal of Cy5 with increasing irradiation time. Carboxy‐terminated NP, as a control, exhibited no decrease in Cy5 fluorescence (Figure S17C, Supporting Information). The photobleaching of [TAT]‐NP can be explained by FRET. The emission spectrum of DEAC is slightly overlapping with the excitation spectrum of Cy5 (Figure 4C). Thus, the energy provided for DEAC excitation is transferred to Cy5, resulting in excitation and gradually causing photobleaching. The calculated photobleaching factor *κ* for an irradiation time of 1 min has a value of 1.496.

### 2.4 In Vitro Performance of NPs

**Figure 5:**
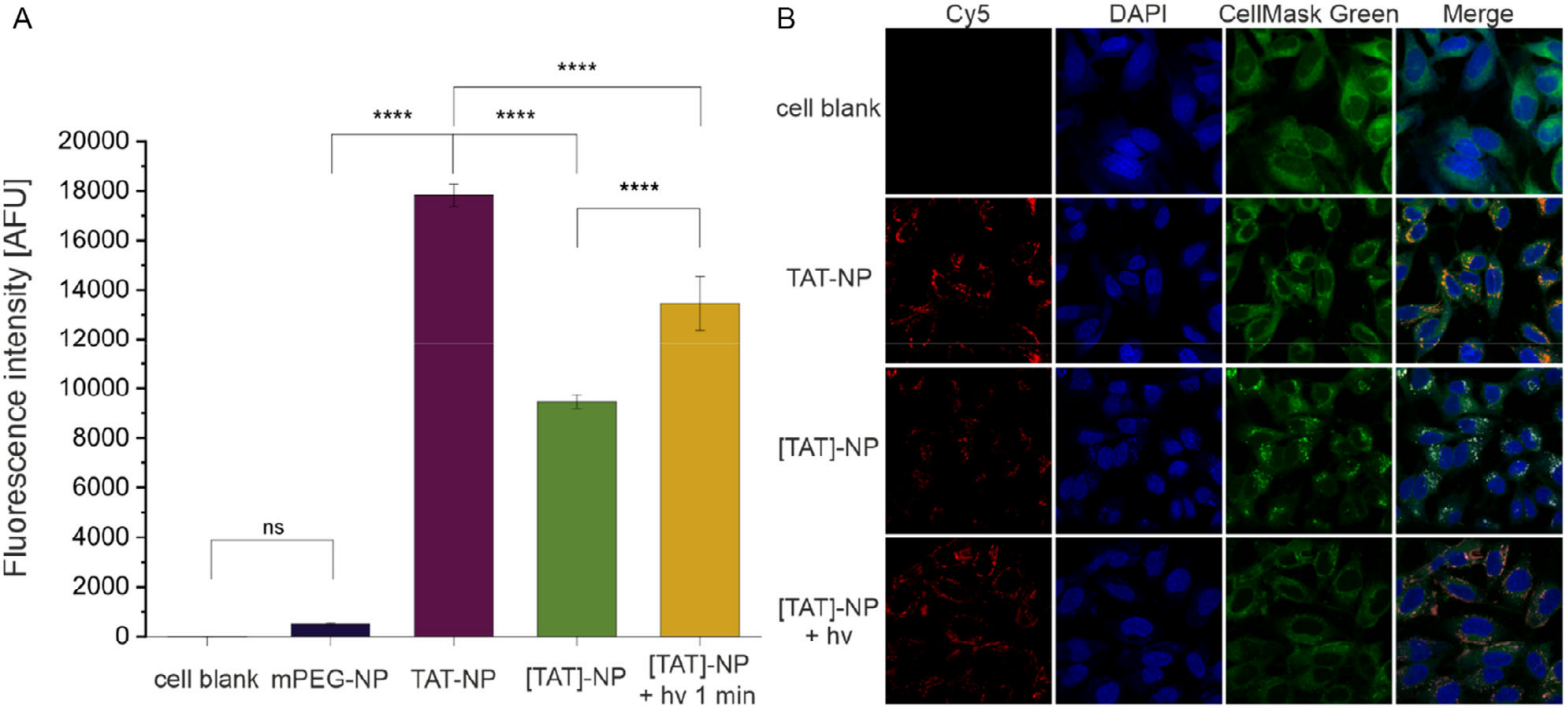
Flow cytometric and CLSM analysis of photo‐responsive NP. A) Results from flow cytometry experiment. Fluorescence intensity of 1 min irradiated [TAT]‐NP ([TAT]‐NP + hv) was multiplied by photobleaching factor *κ*. Data represent mean ± SD (*n *= 3, level of statistical significance is indicated as * *p *≤ 0.05, ** *p *≤ 0.01, *** *p *≤ 0.001, **** *p *≤ 0.0001), (AFU, arbitrary fluorescence units). B) CLSM images showing the cellular uptake of Cy5‐labeled NP (red) in HeLa cells at 24 h postincubation time. Cell nuclei were labeled with DAPI (blue). CellMask Green (green) was used for staining cell membrane and cytosol.

The uptake respectively attachment of mPEG‐, TAT‐, [TAT]‐, and irradiated [TAT]‐NP to HeLa cells were studied by flow cytometry (**Figure** **5**A). Comparing the cell‐associated fluorescence of nonfunctionalized and TAT functionalized NP, functionalized NP showed higher signals. Positively charged TAT‐NP showed a 35‐fold higher uptake in HeLa cells than those exposed to TAT‐free mPEG‐NP. HeLa cells incubated with [TAT]‐NP exhibited less cell‐associated fluorescence in comparison to TAT‐NP, suggesting that the caging strategy prevented CPP‐mediated NP–cell interaction. Measured data of previously irradiated [TAT]‐NP ([TAT]‐NP + hv) was multiplied by photobleaching factor *κ*. The cell associated fluorescence of [TAT]‐NP + hv was greater than the nonirradiated sample ([TAT]‐NP), but less than photocage‐free TAT‐NP. Despite complete reconstitution of zetapotential of masked NP after light irradiation, uptake potential of TAT‐NP could not be completely restored. The incorporation of DEAC into NP design reduced cell associated fluorescence by half, which can also be explained by 56% CE of DEAC to TAT‐PEG_2k_‐PLA_10k_. In addition to noncaged lysine residues, the presence of noncaged arginine residues may contribute to the increased cellular uptake in comparison to blank NP. Theoretically, poly‐lysine peptides might offer an alternative approach to obtain better camouflaged NP. However, their comparatively limited membrane binding and penetrating capabilities reduce their suitability for practical applications.^[^ ^39^ ^]^

**Figure 6:**
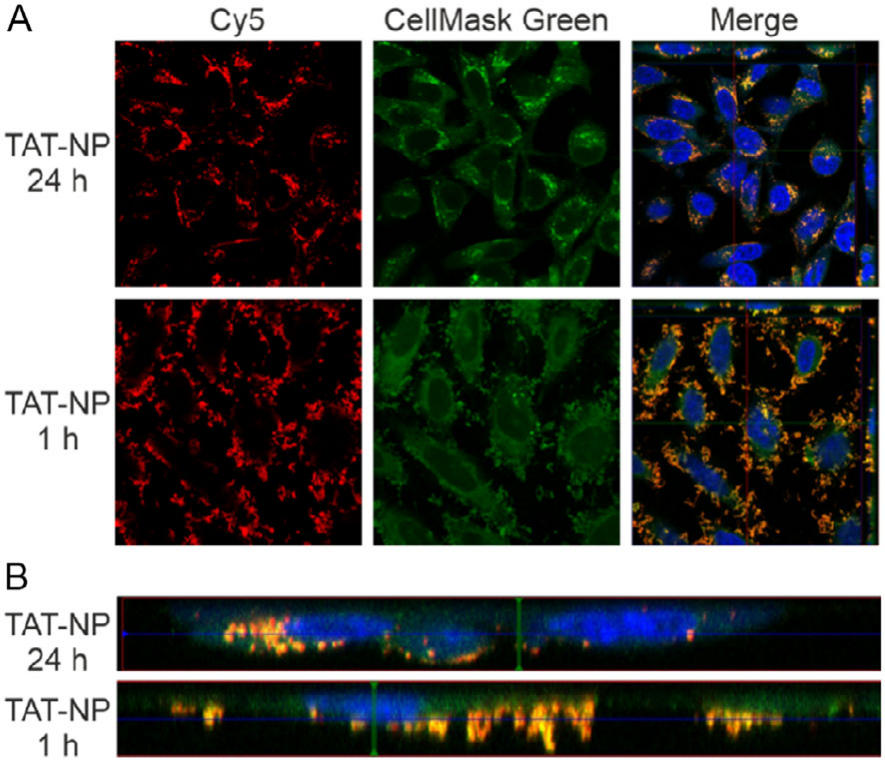
Characterization of interaction between TAT‐NP and HeLa cells. A) CLSM images showing the cellular interaction of TAT‐NP (red) at 24 h and 1 h postincubation time. B) Orthogonal projections of CLSM Z‐stack images enable distinction between surface‐bound and internalized TAT‐NP (red) after different postincubation times. Cell nuclei were labeled with DAPI (blue). Cell membrane and cytosol were stained with CellMask Green (green).

To obtain detailed information at the single‐cell level, confocal microscopy was used. Figure 5B shows the confocal images of treated HeLa cells at 24 h postincubation time. In the merged fluorescence images [TAT]‐NP appeared white. This is caused by the overlap of all three colors as DEAC was excited by laser at 405 and 488 nm while Cy5 was excited 633 nm. Irradiated [TAT]‐NP appear red in the merged channel confirming the absence of DEAC and thus sufficient uncaging. All in all, the findings from flow cytometry were confirmed. To differentiate between cell uptake and adsorption of NP to the cell membrane, cells were investigated after two postincubation times (**Figure** **6**A). Z‐stack images taken at 1 h postincubation time show that NP are attached to cell surface, whereas NP had been taken up by cells 24 h postincubation (Figure 6B).

## 3 Conclusion

We were able to incorporate a PPG into our NP design changing its physicochemical as well as biological properties. While the particles carrying the CPP are genuinely internalized by cells, conjugation of DEAC effectively camouflaged them, thus significantly reducing cell uptake. Upon light irradiation, the CPP‘s penetration enhancing properties were restored. This new NP design offers the possibility to obtain greater spatial and temporal control over NP uptake once they encounter the target cell in a tissue of interest. Due to the absorption properties of the selected coumarin‐derived photocage, which requires activation by light of short‐wavelength with limited tissue penetration, the practical application is limited to tissues that can be directly exposed to light, such as ocular tissues. Recent research from Nizamoglu et al. on optical waveguides for deep‐tissue photomedicine suggests potential for extending applications to surface‐near tissues as well.^[^ ^40^ ^]^ In the future, such NPs could enable a better spatiotemporal targeting of cells in a diseased tissue. Furthermore, they could be used to allow for spatiotemporal drug transfer into target cells.

## 4 Experimental Section

4.1

4.1.1

##### Materials

Ultrapure water was obtained from a Milli‐Q water purification system (Millipore, Schwalbach, Germany). Unless otherwise stated, all chemicals and reagents were obtained from Sigma–Aldrich (Taufkirchen, Germany) in analytical grade. Heterobifunctional poly(ethylene glycol)carboxylic acid with a molecular mass of 2000 g mol^−1^ (COOH‐PEG_2k_‐OH) was purchased from Jemkem Technology USA, Inc. (Allen, TX, USA). Poly(ethylene glycol) methyl ether with a molecular mass of 2000 g mol^−1^ (mPEG_2k_‐OH) as well as Resomer RG 502 H, poly(D, L‐lactide‐co‐glycolide) (lactide:glycolide 50:50, acid terminated, M_*w*_ 7000–1700 Da) (PLGA) were obtained from Sigma–Aldrich (Taufkirchen, Germany). 1‐Ethyl‐3‐(3‐dimethylaminopropyl)carbodiimide (EDC) and 7‐(Diethylamino)‐4‐(hydroxymethyl)coumarin (DEAC) were sourced from TCI (Eschborn, Germany). *N*‐hydroxysuccinimide (NHS) was purchased from Carl Roth (Karlsruhe, Germany). 1‐[Bis(dimethylamino)methylene]1H‐1,2,3‐triazolo[4,5‐b]pyridinium 3‐oxide hexafluorophosphate (HATU) was obtained from Fluorochem (Gadfield, United Kingdom). The fluorescent dye Cy5 was purchased from Lumiprobe (Hannover, Germany). 7‐(Diethylamino)‐4‐methyl‐coumarin, *N*, *N*‐diemthylformaidediemthylacetal (DMFDMA), 4‐nitrophenylchloroformate, and protected amino acids Fmoc‐Arg(Pbf)‐OH, Fmoc‐Gln(Trt)‐OH, Fmoc‐D‐Lys(Boc)‐OH, FmocGly‐OH, and Fmoc‐6‐AHx‐ OH were obtained from BLDpharm (Karlsruhe, Germany). 2‐Chlorotrityl chloride resin was sourced from Carbolution Chemicals (St. Ingbert, Germany). Sodium metaperiodate (NaIO_4_) was purchased from VWR International GmbH (Darmstadt, Germany). The cellulose dialysis membranes had a molecular weight cutoff of 6–8 kDa and were obtained from Spectrum Laboratories, Inc. (Rancho Domingues, CA, USA). The frits had a pore size of 35 µm and were sourced from Roland Vetter Laborbedarf (Ammerbuch, Germany). The infrared lamp was obtained from Medisana (Neuss, Germany), with a thermostat from PEARL GmbH (Buggingen, Germany). Syringes were purchased from Braun (Melsungen, Germany). Nuclear magnetic resonance (NMR) spectra were recorded on a Bruker Avance‐400 or Avance‐500 NMR spectrometer (Bruker, Billerica, MA, USA). High‐resolution mass spectrometry (HRMS) was performed on a Q‐TOF 6540 ultrahigh definition (UHD) liquid chromatography/mass spectrometry (LC/MS) system (Agilent Technologies, Santa Clara, CA, USA) using an electrospray ionization (ESI) source. High‐performance liquid chromatography (HPLC) analysis was performed with 1260 Infinity II from Agilent Technologies (Santa Clara, CA, USA) using a Kinetex EVO C18 column (100 Å, 2.6 µm, 150 mm × 4.6 mm, Phenomenex Ltd. Germany). Preparative HPLC analysis was performed with Prep 150 LC system from Waters (Eschborn, Germany) consisting of a 2545 binary gradient module, a 2489 UV/visible detector and a Waters Fraction Collector III. A Gemini‐NX C18 column (110 Å, 5 µm, 250 mm × 21 mm, Phenomenex Ltd. Germany) was used. Flash chromatography was performed with puriFlashR XS520Plus (Advion Interchim scientific, Ithaka, NY, USA) using prepacked silica cartridges. HeLa (CCL‐2) cells were purchased from ATCC (Manassas, VA, USA) and fetal bovine serum (FBS) for the preparation of cell culture medium was purchased from Biowest (Nuaille, France). For all microscopic experiments, the cells were seeded in 8‐well microscopy slides from Ibidi (Graefelfing, Germany). Dako Faramount Mounting Medium was obtained from Agilent Technologies (Santa Clara, CA, USA). CellMaskTM Green was purchased from Invitrogen, Life Technologies GmbH (Darmstadt, Germany).

##### Synthesis of Cell‐Penetrating Peptide

The TAT sequence was synthesized by manual solid‐phase peptide synthesis using a standard Fmoc strategy following the procedure of Bresinsky et al.^[^ ^41^ ^]^ The 2‐chlorotrityl chloride resin (300 mg, 1.182 mmol g^−1^, 0.355 mmol, 1.00 equiv) was weighed into a fritted 20 mL syringe. 15 mL of dichloromethane (DCM) was added to swell the resin at room temperature (RT) for 30 min. Subsequently, DCM was then aspirated with a vacuum flask. The first amino acid (2.50 equiv) was dissolved in the smallest possible volume of DCM. If this was not feasible, small quantities of dimethylformamide (DMF) were added gradually until complete dissolution was achieved. 2.50 equiv of 2,4,6 collidine was added and the solution was transferred into the syringe and shaken for 3 h at RT. Subsequent to the aspiration of the solvent, the resin containing the bound amino acid was rinsed 3 times with 15 mL DCM. A total of 15 mL of piperidine 20% (V/V) in DMF was drawn into a syringe and agitated on an orbital shaker at 35 °C for 15 min to remove the N‐terminal Fmoc‐protecting group. The shaker was covered with a box insulated with aluminum foil and the temperature was adjusted with an infrared lamp. Temperature was controlled by a thermostat. After deprotection, the residual resin was washed with 15 mL DMF. For the subsequent coupling steps, the respective amino acid (2.50 equiv) and HATU (2.50 equiv) were weighted separately. Both were dissolved in 7 mL, respectively, 5 mL DCM and collidine (2.50 equiv) were added to the solution of HATU. Afterward, both solutions were transferred into the syringe and agitated for 1 h at RT. The deprotecting and coupling steps were repeated until the TAT sequence was built up. Finally, the resin was rinsed with methanol, DCM, diethyl ether, each (2 × 15 mL), using a vacuum flask. For peptide detachment from resin, the dried resin was transferred into a round‐bottom flask and a solution of hexafluoroisopropanol 20% (HFIP) in DMF was added dropwise. After stirring for 2 h, the solution was filtrated and the filtrate was evaporated. Then, the sidechain protected peptide was analyzed via HPLC and mass spectrometry. For further detail see Supporting Information, Table S1.

##### Polymer Synthesis

COOH‐PEG_2k_‐PLA_10k_ and mPEG_2k_‐PLA_10k_ block copolymers were synthesized according to Qian et al. with modifications as previously described by our group.^[^ ^42^ ^]^ In brief, the heterobifunctional PEG polymer (1 equiv.), COOH‐PEG_2k_, respectively, mPEG_2k_, served as macroinitiator for the reaction with cyclic lactide (3,6‐dimethyl‐1,4‐dioxane‐2,5‐dione) (70 equiv.). Both were dissolved in anhydrous DCM and mixed with 1,8‐diazabicyclo [5.4.0]undec‐7‐ene (DBU) (3 equiv.). The mixture was stirred for precisely 1 h at RT. Subsequently, the polymerization reaction was quenched with benzoic acid (10 equiv.). The reaction product was precipitated in 20‐fold amount of ice‐cold diethyl ether and dried under nitrogen flow overnight at RT. The resulting polymers were characterized via ^1^H‐NMR.

##### Fluorescence Labeling of PLGA

To detect NPs in cells, the core forming PLGA was covalently linked to a fluorescent dye as previously described by our group.^[^ ^43^ ^]^ In brief, carboxylic acid‐terminated PLGA (Resomer RG 502 H) (1 equiv.) and cyanine‐5‐amine (0.1 equiv.) were dissolved in anhydrous DMF. For activation of the carboxylic acid 2‐(1H‐benzotriazol‐1‐yl)−1,1,3,3‐tetramethyluronium‐hexafluorophosphat (HBTU) (2 equiv.) was added to the reaction mixture. Subsequently, *N*‐ethyl‐*N*‐(propan‐2‐yl)propan‐2‐amine (DIPEA) (4 equiv.) was added and the reaction mixture was stirred overnight at RT under light exclusion. The excess of free fluorescent dye was removed by precipitation in 20‐fold amount of ice‐cold diethyl ether and dried under nitrogen. The polymer was dissolved in ACN and precipitated again. This step was repeated until the supernatant was no longer blue. The product was dried under nitrogen flow at RT.

##### Synthesis of 7‐Diethylamino‐4‐Hydroxymethylcoumarin (DEAC)

The PPG DEAC was synthesized as previously described by Weinrich et al. with small modifications.^[^ ^44^ ^]^ Synthesis scheme is depicted in Figure 1B. For detailed synthesis see Supporting Information.

##### Activation of DEAC

For synthesis of caged TAT ([TAT]) the photocage DEAC needed to be activated. Therefore, the hydroxy function was transformed into a reactive carbonate. A solution of DEAC **4** (234 mg, 946 µmol, 1.00 equiv.) and 4‐nitrophenylchloroformate (1.91 g, 9.47 mmol, 10.0 equiv.) in anhydrous DCM (25 mL) was cooled to 0 °C. DIPEA (1.65 mL, 9.47 mmol, 10.0 equiv.) was added dropwise and stirred for 15 min. The reaction solution was then stirred for 6 h at RT. Subsequently, the mixture was washed twice with 0.01 N HCl 100 mL. Organic layer was removed under reduced pressure. Purification of synthesis product was performed by preparative HPLC. The mobile phase consisted of solvent A (Millipore water with 0.1%) and solvent B (acetonitrile). The separation was initiated with an isocratic hold at 20% B for 1 min, followed by a linear gradient from 20% to 95% B over 19 min at a flow rate of 20 mLmin^−^^1^. Detection was performed at 220 nm. Fractions containing the desired product were collected, pooled, and lyophilized to yield the purified compound.

##### TAT 48−57 Coupling to Polymer

For activation of block copolymer, COOH‐PEG_2k_‐PLA_10k_ was dissolved in anhydrous DMF and EDC and NHS (each 25 equiv.) were added as powder. The mixture was stirred for 1 h at RT. β‐mercaptoethanol (35 equiv.) were added for 15 min at RT to quench the excess of EDC. The protected TAT 48−57 was dissolved in 1 mL DMF and added to the reaction mixture. DIPEA (10 equiv.) was added and the reaction was stirred for 24 h at RT. Subsequently, the reaction product was precipitated in 20‐fold amount of ice‐cold diethyl ether and dried under nitrogen flow overnight at RT. Deprotection of all sidechains of the TAT 48–57 was performed after the coupling reaction to enable a specific N‐terminus coupling to the polymer. The modified polymer was dissolved in a mixture of 3 mL trifluoroacetic acid, phenol, water, triisopropylsilane (88/5/5/2 [%]) and stirred for 1 h at RT. Afterward, the reaction product was precipitated in 20‐fold amount of ice‐cold diethyl ether and dried under vacuum overnight. The reaction product was dissolved in as little ACN as possible. Small quantities of dimethyl sulfoxide were used as solubilizer. Polymeric micelles were produced by dropwise addition of polymer solution into a 10‐fold excess of vigorously stirring Millipore water. The solution was stirred for 3 h under a fume hood to evaporate the organic solvent. The excess of TAT 48−57 and regents were removed by dialysis. Polymeric micelles were transferred into a dialysis tube with a molecular weight cutoff of 6–8 kDa against 4 L of Millipore water with medium change after 30 min, 2 h and 14 h; 19 h total. The polymeric micelles were lyophilized to obtain TAT48‐57‐PEG_2k_‐PLA_10k_ (TAT‐PEG_2k_‐PLA_10k_). The CE was determined by Fluram assay.

##### Fluram Assay

Initially, the amount of lysine hydrochloride corresponding to 100% CE was calculated (see Supporting Information, Equation 7). A calibration line was recorded in order to verify the linear relationship between free primary amine and fluorescence signal. Therefore, lysine hydrochloride stock solution (2 mg mL^−1^ in Millipore water) was diluted to reach a concentration range of 5–40 µg mL^−1^. Micelles of TAT‐PEG_2k_‐PLA_10k_ were formed by nanoprecipitation. TAT‐PEG_2k_‐PLA_10k_ stock solution was diluted to a concentration of 10 mg mL^−1^ and dropwise added into 10‐fold amount of Millipore water with vigorous stirring. The solution was stirred for 3 h under a fume hood to evaporate the organic solvent. Fluorescamine reagent was freshly prepared by dissolving fluorescamine in acetone to reach a final concentration of 0.3 mg mL^−1^. The assay was performed by pipetting 170 µL borate buffer (pH 8.5) into a white 96‐well plate. Subsequently, 10 µL of micelles or lysine hydrochloride was added on top and the well plate was shaken for 5 min at 150 rpm. The pH was checked to be at 8.5. Afterward, 20 µL fluorescamine reagent was added to the mixture and incubated at RT for 1 min while shaking at 150 rpm. The fluorescence was measured at *λ*_em _= 485 nm (*λ*_ex _= 380 nm) on a FLUOstar Omega plate reader (BMG Labtech, Ortenberg, Germany). The CE was determined by setting the measured value of TAT micelles in relation to the amount of lysine hydrochloride which corresponds to 100% CE.

##### Synthesis of Caged Polymer [TAT]‐PEG_2k_‐PLA_10k_

TAT‐PEG_2k_‐PLA_10k_ (80 mg, 5.91 µmol, 1.00 equiv.) was dissolved in the smallest possible amount of DMF. Subsequently, DIPEA (15.3 µL, 87.8 µmol, 14.8 equiv.) and activated DEAC (18.14 mg, 44.03 µmol, 7.44 equiv.) were added to the solution and stirred for 6 days at RT. Subsequently, the reaction product was precipitated in 20‐fold amount of ice‐cold diethyl ether and dried under nitrogen flow overnight at RT.

##### Quantification of Caged Polymer

DEAC (10 mg mL^−1^) and [TAT]‐PEG_2k_‐PLA_10k_ (40 mg mL^−1^) were dissolved in ACN. DEAC was diluted to achieve a concentration range of 2.5–20 µg mL^−1^ for construction of a calibration line. [TAT]‐PEG_2k_‐PLA_10k_ was diluted to 400 µg mL^−1^. Absorption spectra were recorded and absorbance was measured at *λ*_ex _= 377 nm on a FLUOstar Omega plate reader. The absorption was used to calculate CE of DEAC to TAT polymer (see Supporting Information, Equation 11).

##### Spectral Characterization of DEAC and DEAC Conjugates

Absorption measurements were performed using a FLUOstar Omega plate reader. Therefore, 200 µL sample were transferred into quartz 96‐well plate (Hellma GmbH & Co. KG, Müllheim, Germany). Fluorescence spectra were obtained by Cary Eclipse or BioTek Synergy Neo2 (Agilent Technologies, Santa Clara, CA, USA) using a quartz cuvette or black well plate.

##### Photocleavage of DEAC

For all uncaging experiments a LED (LED‐TECH.DE optoelectronics GmbH, Moers, Germany) with *λ*_em _= 365 nm and lens SM2F (Thorlabs Inc., NJ, USA) were used (see Figure S17 A, Supporting Information). The LED irradiance power was measured with FieldMax II Top laser power and energy meter (Coherent, Saxonburg, PA, USA). The sample was transferred into a microUV‐transparent cuvette (Brand GmbH+Co KG, Wertheim, Germany) and irradiated with different powers and over different times.

##### Cleavage of DEAC‐NH‐PEG_2k_‐PLA_10k_

In order to determine irradiation time and energy required for photocleavage of DEAC, DEAC‐NH‐PEG_2k_‐PLA_10k_ was irradiated under various conditions. The uncaging was monitored via HPLC. The mobile phase consisted of solvent A (Millipore water with 0.05% trifluoroacetic acid) and solvent B (acetonitrile with 0.05% trifluoroacetic acid). The flow rate was maintained at 1 mLmin^−1^ The chromatographic method initiated with an isocratic hold at 95% A/5% B for 1 min, followed by a linear gradient to 5% A/95% B over 15 min. The high‐organic phase was maintained for 4 min, after which the system was returned to the initial conditions (95% A/5% B) for column re‐equilibration. Detection was carried out a fluorescence detector with excitation at 375 nm and emission monitored at 480 nm.

##### Uncaging of [TAT]‐Nanoparticles

The cleavage of DEAC from [TAT]‐NP was studied by zetapotential measurements. First, NPs were irradiated for 1, 2 and 5 min with a LED power of 100 mW. Subsequently, NPs were diluted 1:20 in 2 mm NaCl_2_ and then zetapotential was measured.

##### Photobleaching of Cy5

Photobleaching of Cy5 was observed during uncaging experiments. Therefore, further investigations on quantification of photobleaching were performed. Carboxy‐terminated NP (COOH‐NP) as well as [TAT]‐NP were irradiated for various time points with a LED power of 100 mW. Afterward, NPs were diluted 1:4 with Millipore water and fluorescence intensity of Cy5 (*λ*_ex _= 640 nm, *λ*_em _= 681 nm) was measured. A photobleaching correlation factor *κ* was calculated (Equation 1).

(1) $$\text{κ}=\frac{RFU_{no\text{irradiation}}}{RFU_{60s}}$$

RFU is defined as relative fluorescence units.

##### Nanoparticle Preparation and Characterization

Core–shell NPs were manufactured by nanoprecipitation. PLGA and the corresponding PLA‐PEG copolymer were dissolved at a ratio of 30/70 (m/m) in ACN reaching a final concentration of 10 mg mL^−1^. The organic phase was added dropwise into the 10‐fold excess of stirring (900 rpm) 10% Dulbecco's phosphate‐buffered saline (DPBS). The mixture was stirred for 3 h at RT to remove the organic solvent.

NP with methoxy‐terminated polymer chains (mPEG_2k_‐PLA_10k_) served as a negative control, whereas NP consisting of 50% TAT‐PEG_2k_‐PLA_10k_ and 50% mPEG_2k_‐PLA_10k_ served as a positive control.

NTA (NanoSight NS300, Malvern, United Kingdom) was used to determinate nanoparticle size (hydrodynamic diameter (d_*h*_)) and concentration. For NTA measurements the NP were diluted with Millipore water to reach a particle concentration of 20–100 particles per frame. The size distribution (PDI) was calculated with the following equation.

(2) $$\mathrm{PDI}={\left(\frac{\sigma }{\mu }\right)}^{2}$$

*µ* is defined by the mean particle size and *σ* is representing the standard deviation.

Zetapotential measurements were performed with Malvern Zetasizer Nano ZS (Malvern, United Kingdom). Therefore, NP were diluted with 2 mm NaCl_2_ by a factor of 20.

##### Cell Culture

HeLa cells were cultivated in Eagle's minimum essential medium (EMEM) and with 10% FBS (PAN‐ biotech GmbH, Aidenbach, Germany).

##### Flow Cytometry

HeLa cells were seeded into 24‐well plates at a concentration of 80,000 cells/well and incubated for 24 h at 37 °C and 5% CO_2_. NPs containing Cy5‐PLGA were prepared and characterized. [TAT]‐NP were uncaged by irradiation with LED for 1 min at 100 mW and zetapotential was measured as described before. Leibovitz medium (LM) was used to adjust the NP concentration to 100 pM. The cell medium was discarded and 200 µL of NP formulation was added to each well. LM was used as cell blank. After incubation for 60 min at 37 °C, 5% CO_2_, the particle solution was discarded, washed with 500 µL warm PBS and the cells were harvested using 300 µL of 0.05% typsin/0.02% EDTA solution. All subsequent work was done on ice. After complete cell detachment, typsin was quenched by the addition of 400 µL EMEM + 10% FBS and the cell suspension of each well was transferred into Eppendorf Tubes. After centrifugation for 5 min at 200 rcf at 4 °C, the supernatant was aspirated and the cell pellets were washed with 500 µL cold PBS. Centrifugation step was repeated and the cell pellets were resuspended in 300 µL cold PBS and stored on ice. Directly before the flow cytometry measurement, the cell suspension was transferred into flow cytometry tubes. The samples were analyzed using a FACS Canto II (Becton Dickinson, Franklin Lakes, NJ). NPs were excited at 633 nm, and the emission was recorded using a 661/16 nm bandpass filter. Flow cytometry data was analyzed using Flowing software 2.5.1 (Turku Centre for Biotechnology, Turku, Finland, with the support of Biocenter Finland). To take photobleaching of Cy5 into account, the results from [TAT]‐NP were multiplied by an experimentally determined correlation factor *κ*.

##### Confocal Laser Scanning Microscopy (CLSM)

For a detailed analysis of NP–cell interaction, HeLa cells were seeded into 8‐well Ibidi slide at a concentration of 20,000 or 10,000 cells/well and cultured for 24 h, respectively, 48 h at 37 °C, 5% CO_2_. NP concentration was adjusted to 100 pM by dilution with LM. The cell medium in the wells was then replaced by 200 µL of NP formulation. LM was used as cell blank. After incubation for 60 min at 37 °C, 5% CO_2_, the particle solution was aspirated and cells were washed with 300 µL prewarmed sterile PBS. Then, 300 µL medium was added and the cells were incubated for further 24 h, respectively, 1 h. Afterward, the medium was aspirated and the cells were incubated with 180 µL Cell Mask Green staining solution (5 µg mL^−1^ in LM) for 10 min at 37 °C, 5% CO_2_ for membrane and cytosol staining. The cells were washed twice with 300 µL prewarmed PBS. Then, fixation of the cells was conducted by adding 250 µL of 4% paraformaldehyde (PFA) solution in PBS. The cells were incubated for 15 min at RT and then washed with 300 µL prewarmed PBS. Subsequently, the cell nuclei were stained by incubating 4′,6′‐diamidine‐2′‐phenylindole dihydrochloride (DAPI) staining solution (1 µg mL^−1^ in PBS) for 10 min at RT. Following an additional washing step, the cells were mounted using Dako Farmount Mounting Medium and stored at 37 °C in the dark until measurement. The cells and NPs were imaged using Zeiss LSM 710 confocal microscope (Carl Zeiss microscopy GmbH, Jena, Germany). Fluorescence signals were acquired in separate channels using dye‐specific excitation lasers: 405 nm for DAPI, 488 nm for CellMask Green, and 633 nm for Cy5. These individual channels were subsequently combined to merged images. Furthermore, images were taken at different focal planes to create a Z‐stack and obtain 3D information of the sample. Images were analyzed using Zeiss Zen software 3.8.

##### Transmission Electron Microscopy

For imaging of mPEG‐, TAT‐, and [TAT]‐NP, particles were prepared as outlined above. Subsequently, 5 µL of NPs were transferred on a 9 nm carbon film, which had been floated onto a 3.05 mm Cu‐TEM grid (400 mesh) for 5 s at RT. The sample was removed with tissue paper and applied 3 more time in case of mPEG‐NP and then washed with bidistilled water. Uranyl acetate (1% in demineralized water, 5 µL, 30 s at RT) was used for staining. Images were captured at a magnification of 200,000‐ fold on a JEM2100F instrument (JEOL, Freising, Germany) using SerialEM software. For analysis Fiji software was used.^[^ ^45^ ^]^ Visualization and statistical analysis of data was performed with R software 4.3.2 (R Core Team (2022). R: A language and environment for statistical computing. R Foundation for Statistical Computing, Vienna, Austria. URL https://www.R‐project.org/).

##### Statistics

One‐way ANOVA was used to assess group differences. Brown–Forsythe test was applied to verify homogeneity of variances. If ANOVA indicated significance (*p  *< 0.05), Tukey's post hoc test was used for pairwise comparisons. Analyses were performed using Origin 2002b (OriginLab Corporation, Northampton, MA, USA) software, with a significance set at *p *< 0.05.

## Conflict of Interest

The authors declare no conflict of interest.

## Author Contributions

**Jan Birringer**: conceptualization (lead); formal analysis (lead); investigation (lead); methodology (lead); visualization (lead); writing—original draft (lead). **Johannes Konrad**: formal analysis (supporting); methodology (supporting). **Stephan Melchner**: investigation (supporting); methodology (supporting). **Marius Remmert**: investigation (supporting); methodology (supporting). **Achim Goepferich**: conceptualization (lead); funding acquisition (lead); methodology (lead); resources (lead); supervision (lead); writing—review & editing (lead).

## Supporting information

Supplementary Material

## Data Availability

The data that support the findings of this study are available from the corresponding author upon reasonable request.
