# Supplementary material for: Coumarin‐Caged Nanoparticle for Light‐Driven Surface Modification
Source: ChemMedChem. 2025 Oct 7;20(22):e202500636. doi: 10.1002/cmdc.202500636 (PMC12640661; doi:10.1002/cmdc.202500636)
Supplement: Supplementary file 1 — Supplementary Material [file CMDC-20-e202500636-s001.pdf]

Supporting Information for

Coumarin-caged Nanoparticle for Light-driven Surface Modification

Jan Birringer[1], Johannes Konrad[1], Stephan Melchner[1], Marius Remmert[1], Achim Goepferich[1]*

[1]Department of Pharmaceutical Technology, University of Regensburg, 93053 Regensburg, Bavaria, Germany.

*Achim Goepferich
Email: achim.goepferich@ur.de
Phone: +49 941 943-4843

1 Synthesis and Characterization

1.1 Polymer Synthesis

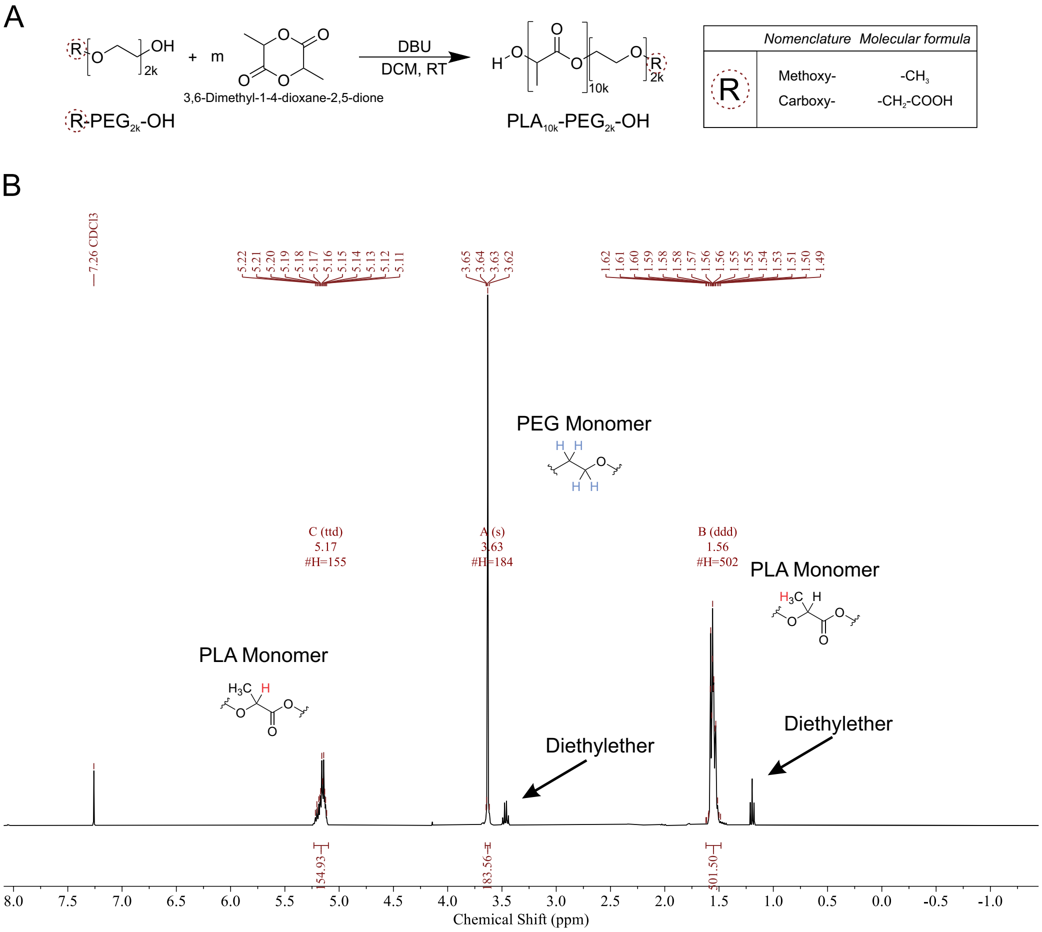

Figure S1: Block copolymer synthesis and characterization. **A** Chemical equation for the synthesis of PLA-PEG block copolymer. The block copolymers were synthesized with different end groups (R). **B** Exemplary ^{1}H-NMR spectrum (400 MHz, $CDCl_3$) of COOH-PEG$_{2k}$-PLA$_{10k}$ block copolymer.

1.2 Synthesis of protected TAT 48-57$_{prot.}$

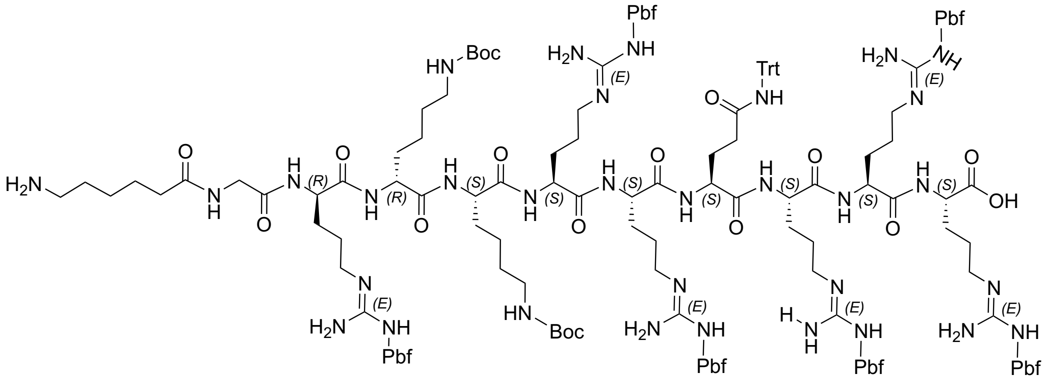

Chemical Formula: $C_{168}H_{246}N_{32}O_{35}S_6$
Exact Mass: 3463,68
Molecular Weight: 3466,37

Figure S2: Structure of TAT 48-57$_{\text{prot.}}$.

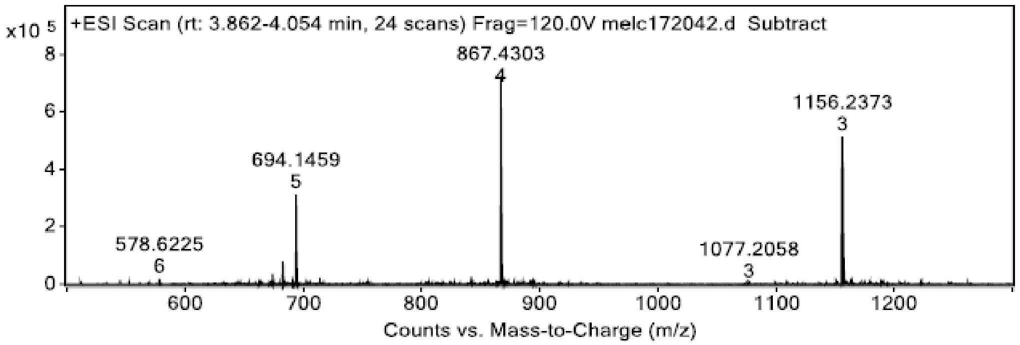

Figure S3: Mass spectrum of TAT 48-57$_{\text{prot.}}$.
HRMS (ESI) m/z calculated for $C_{168}H_{246}N_{32}O_{35}S_6 + 4H^+$: 867.4279, found: 867.4303

**Time [min]**	H_2O + 0.05 %TFA	ACN + 0.05 %TFA
0	95	5
1	95	5
16	5	95
22	5	95
22.30	95	5
30	95	5

Table S1: HPLC method for analysis of TAT 48-57$_{\text{prot.}}$. Flow rate: 1 mL

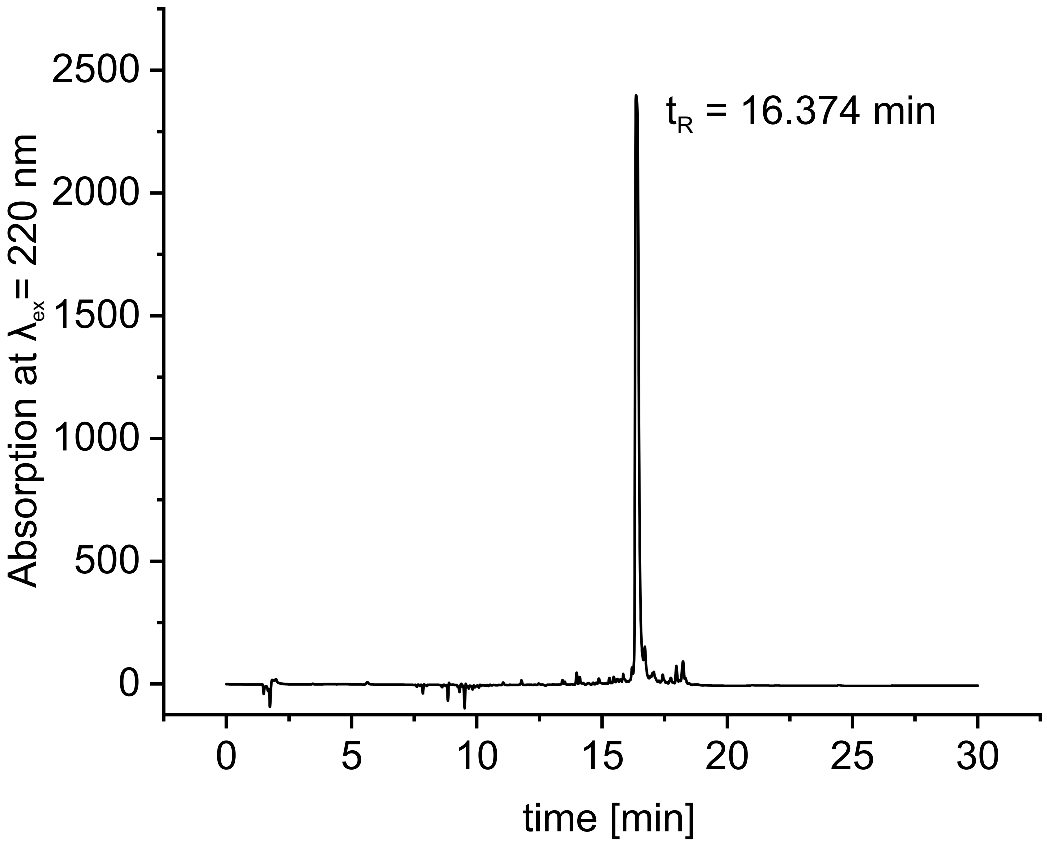

Figure S4: RP-HPLC analysis of TAT 48-57$_{\text{prot.}}$. Retention time t_R of TAT 48-57$_{\text{prot.}}$: 16.374 min

1.3 Synthesis of 7-diethylamino-4-hydroxymethylcoumarin (DEAC)

7-(diethylamino)-4-(2-dimethylamino)vinylcoumarin (2)

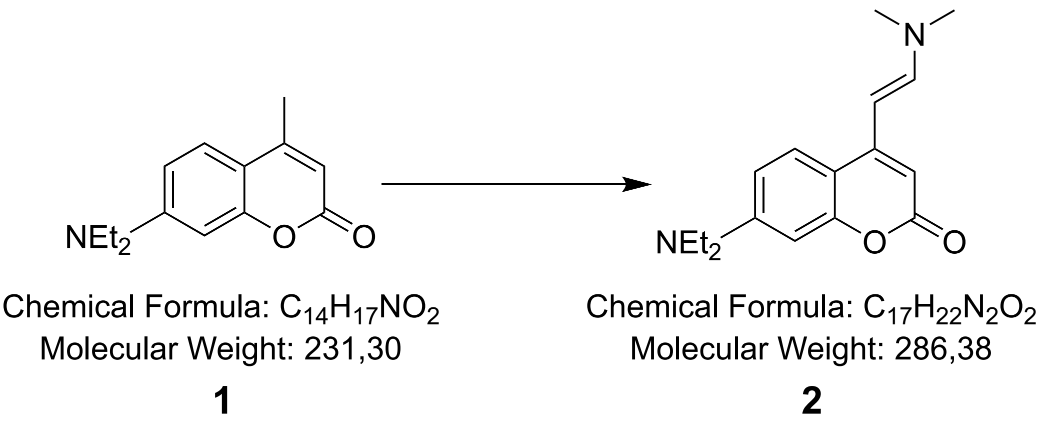

Scheme S1: Reaction equation for synthesis of 7-(diethylamino)-4-(2-dimethylamino)vinylcoumarin (**2**)

To a solution of 7-(diethylamino)-4-methyl-coumarin **1** (24.2 g, 105 mmol, 1.00 equiv.) in DMF (100 mL) DMF–DMA (25 mL, 210 mmol, 2.00 equiv.) was added. The reaction mixture was heated to reflux for 22 h. Reaction was cooled to RT and subsequently conc. $NaHCO_3$ solution and CH_2Cl_2 were added. The organic layer was separated and the aqueous layer was extracted with CH_2Cl_2. Organic layers were combined and solvent was removed under reduced pressure. Purification of synthesis product by flash chromatography gave title compound **(2)** as a goldish solid. Mobile phase: linear gradient, 0-10 min: petrol ether/ethyl acetate 80:20-40:60, 10-15 min: 40:60 (isocratic), 15-19:30 min: 40:60-20:80, 19:30-25 min: 20:80-0:100, 25-70 min: 0:100 (isocratic). Flow: 50 mL/min. Detection was performed at 366 nm.

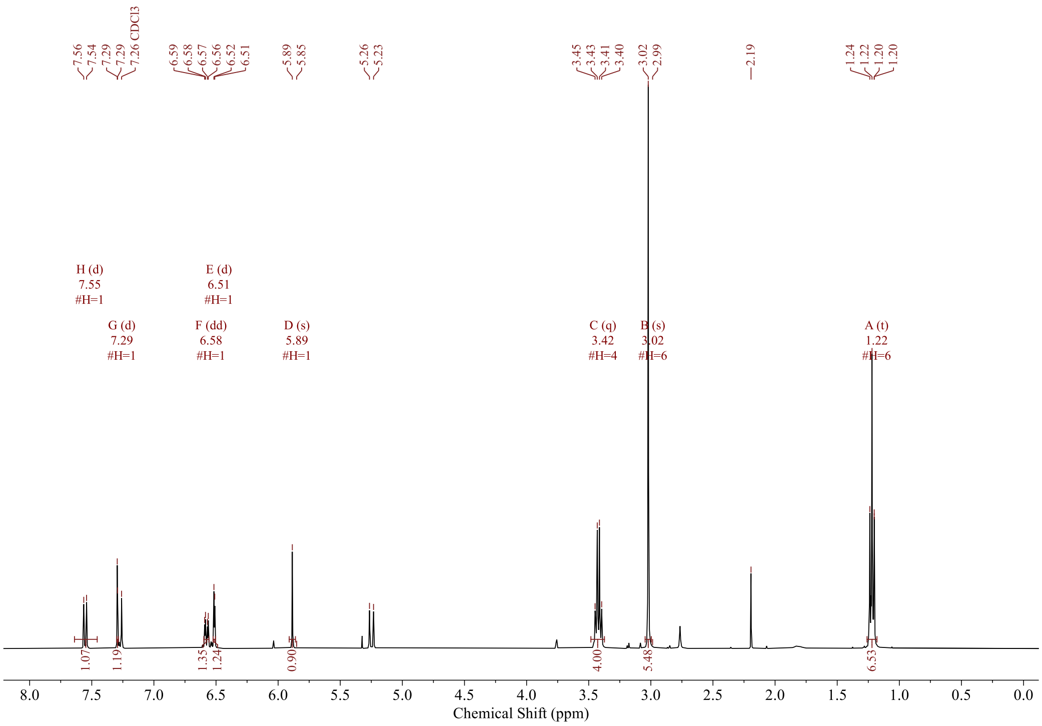

Figure S5: ^{1}H-NMR spectrum of 7-(diethylamino)-4-(2-dimethylamino)-vinylcoumarin. ^{1}H-NMR (400 MHz, $CDCl_3$) δ(ppm) 7.55 (d, J = 9.1 Hz, 1H), 7.29 (d, 1H), 6.58 (dd, J = 9.0, 2.6 Hz, 1H), 6.51 (d, J = 2.6 Hz, 1H), 5.89 (s, 1H), 3.42 (q, J = 7.1 Hz, 4H), 3.02 (s, 6H), 1.22 (t, J = 7.0 Hz, 6H).

7-diethylamino-4-formylcoumarin (3)

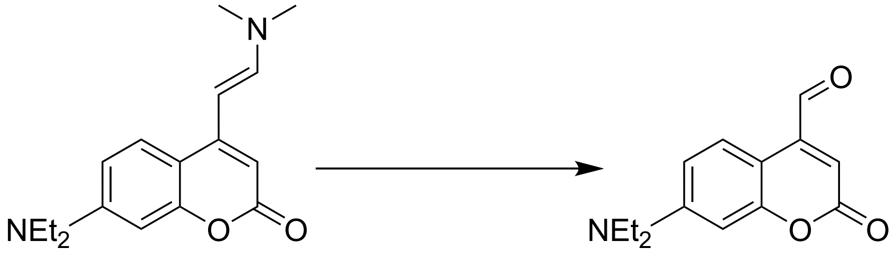

Chemical Formula: $C_{17}H_{22}N_2O_2$
Molecular Weight: 286,38

2

Chemical Formula: $C_{14}H_{15}NO_3$
Molecular Weight: 245,28

3

Scheme S2: Reaction equation for synthesis of 7-diethylamino-4-formylcoumarin (**3**)

The enamine **2** (3.74 g, 13.1 mmol, 1.00 equiv.) was suspended in a mixture of THF/DCM (110 mL 11:1). A solution of $NaIO_4$ (8.40 g, 39.2 mmol, 3.00 equiv.) in water was added and the resulting mixture was stirred at RT for 2 h. The mixture was filtered on Celite and washed with ethyl acetate. Half of the solvent was removed under reduced pressure and conc. $NaHCO_3$ solution was added. The organic layer was separated and the aqueous layer was extracted with CH_2Cl_2. The combined organic layers were dried with $MgSO_4$ and the solvent was removed under reduced pressure. Purification by silica gel chromatography (ethyl acetate/petrol ether, 1:1) gave title compound **(3)** (2.73 g, 85.0 %) as a redish solid.

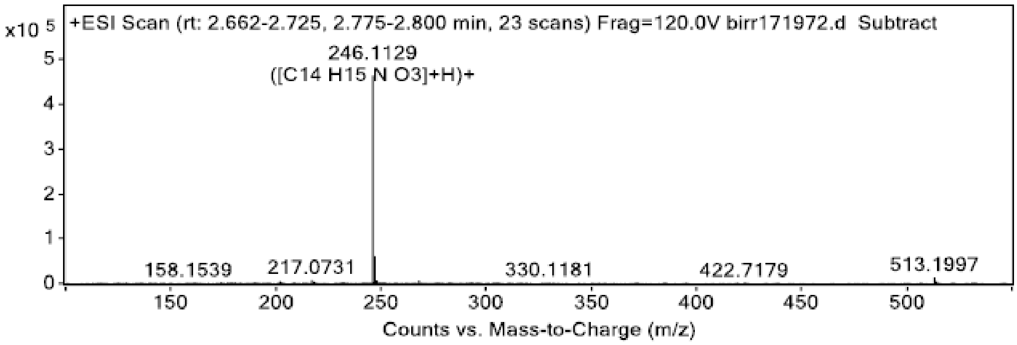

Figure S6: Mass spectrum of 7-diethylamino-4-formylcoumarin$_{\text{prot.}}$.
MS (ESI) m/z calculated for $C_{14}H_{15}NO_3 + H^+$: 246.11, found: 246.1129.

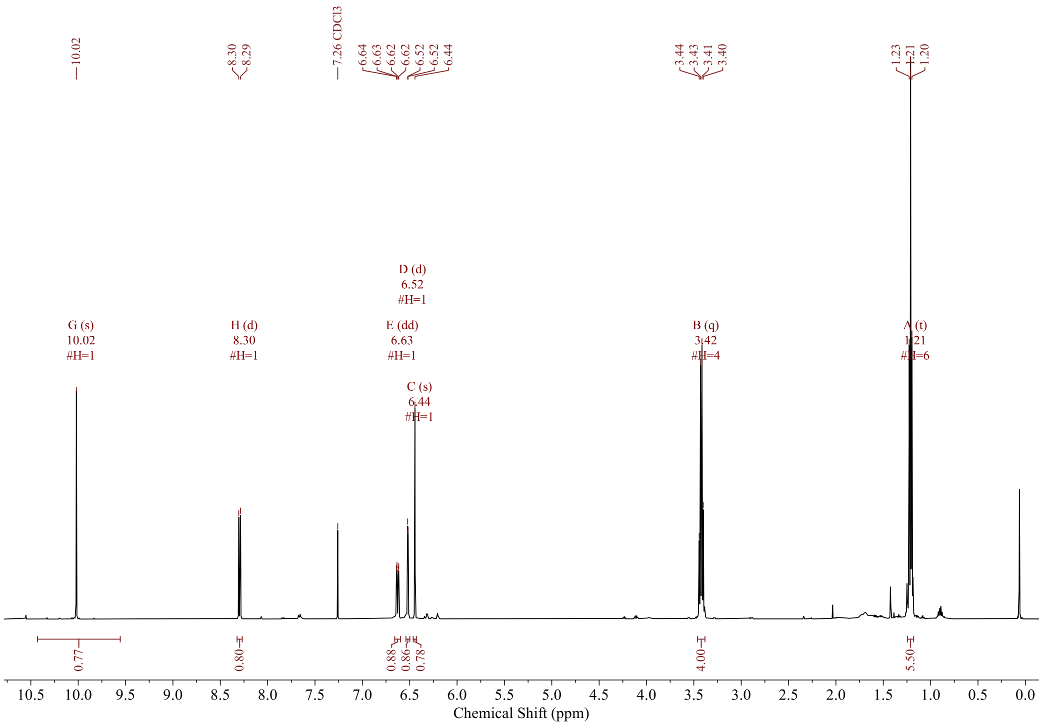

Figure S7: ^{1}H-NMR spectrum of 7-diethylamino-4-formylcoumarin. ^{1}H-NMR (500 MHz, $CDCl_3$) δ(ppm) 10.02 (s, 1H), 8.30 (d, J = 9.2 Hz, 1H), 6.63 (dd, J = 9.2, 2.6 Hz, 1H), 6.52 (d, J = 2.6 Hz, 1H), 6.44 (s, 1H), 3.42 (q, J = 7.1 Hz, 4H), 1.21 (t, J = 7.1 Hz, 6H).

7-diethylamino-4-hydroxymethylcoumarin (4)

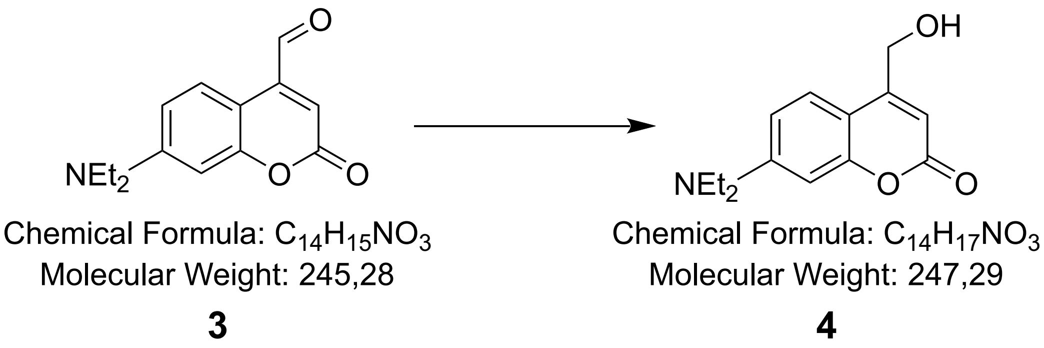

Scheme S3: Reaction equation for synthesis of 7-diethylamino-4-hydroxymethylcoumarin (**4**)

A solution of aldehyde **3** (2.63 g, 10.7 mmol, 1.00 equiv.) in THF (50 mL) was cooled to 0 °C. $NaBH_4$ (984 mg, 21.4 mmol, 2.43 equiv.) was added and the mixture was stirred for 5 h at RT. Subsequently conc. $NaHCO_3$ solution was added, the organic layer was separated and the aqueous layer was extracted with CH_2Cl_2. Title compound **4** (2.46 g, 92.7 %) was isolated as a yellow solid.

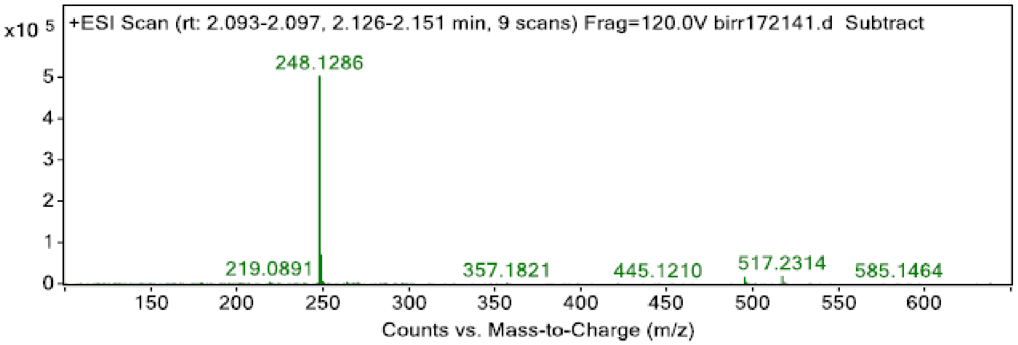

Figure S8: Mass spectrum of 7-diethylamino-4-hydroxymethyl-coumarin$_{\text{prot.}}$.

MS (ESI) m/z calculated for $C_{14}H_{17}NO_3 + H^+$: 248.12, found: 248.1286.

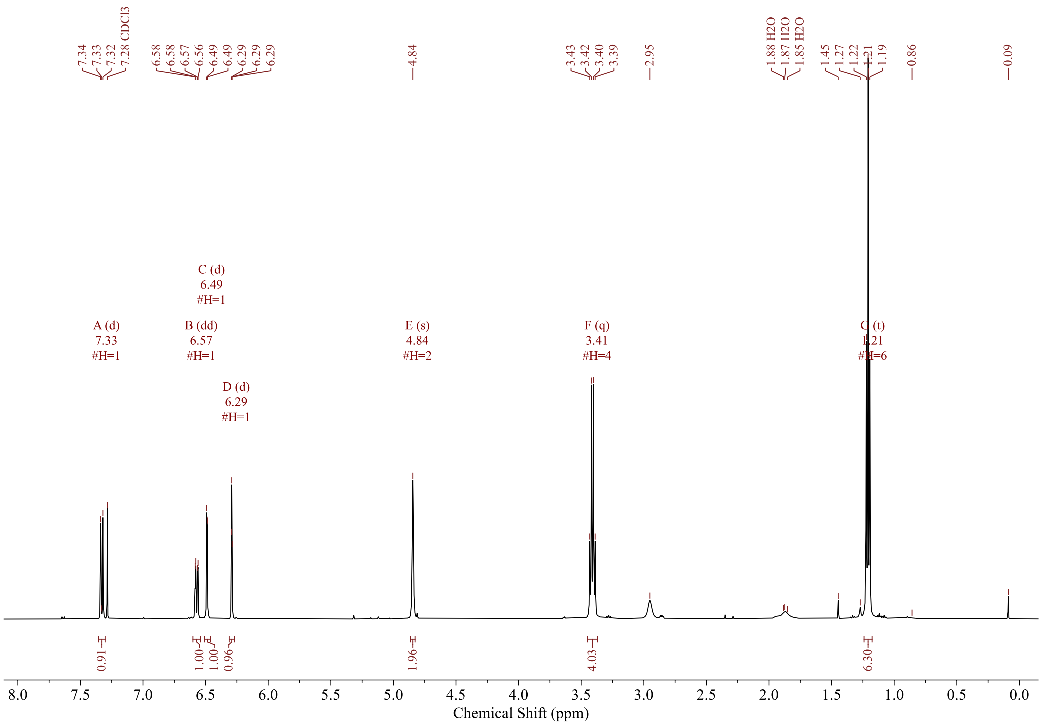

Figure S9: ^{1}H-NMR spectrum of 7-diethylamino-4-hydroxymethyl-coumarin. ^{1}H-NMR (500 MHz, $CDCl_3$) δ(ppm) 7.33 (d, J = 9.0 Hz, 1H), 6.57 (dd, J = 9.0, 2.6 Hz, 1H), 6.49 (d, J = 2.6 Hz, 1H), 6.29 (d, J = 1.4 Hz, 1H), 4.84 (s, 2H), 3.41 (q, J = 7.1 Hz, 4H), 1.21 (t, J = 7.1 Hz, 6H).

**Time [min]**	H_2O + 0.05 %TFA	ACN + 0.05 %TFA
0	75	25
1	75	25
21	40	60
21.1	5	95
25	5	95
25.1	75	25
30	75	25

Table S2: HPLC method for analysis of 7-diethylamino-4-hydroxymethyl-coumarin. Flow rate: 1 mL

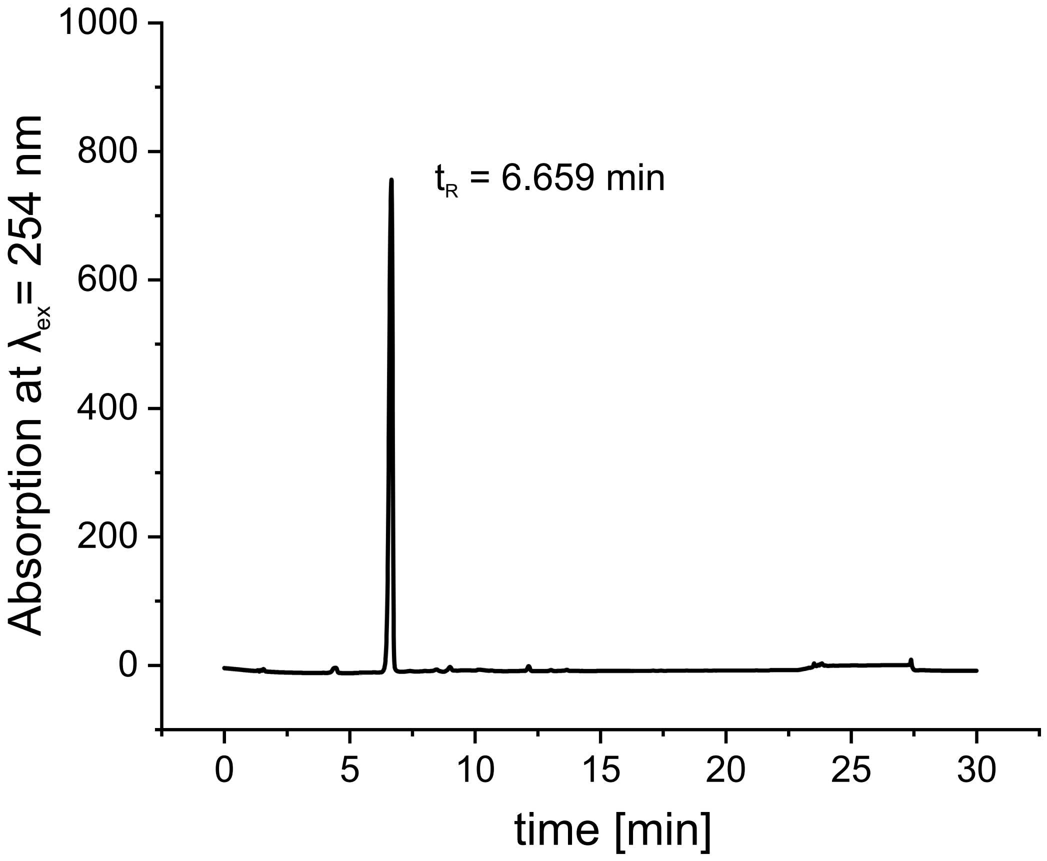

Figure S10: RP-HPLC analysis of 7-diethylamino-4-hydroxymethyl-coumarin. Retention time t_R : 6.659 min. Purity of 91.99 %.

1.4 Activation of DEAC

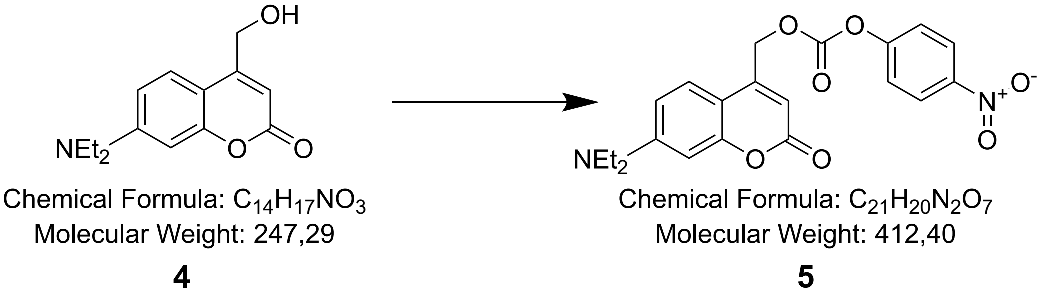

Scheme S4: Reaction equation for synthesis of 7-(diethylamino)-2-oxo-2H-chromen-4-yl)methyl (4-nitrophenyl) carbonate (**5**)

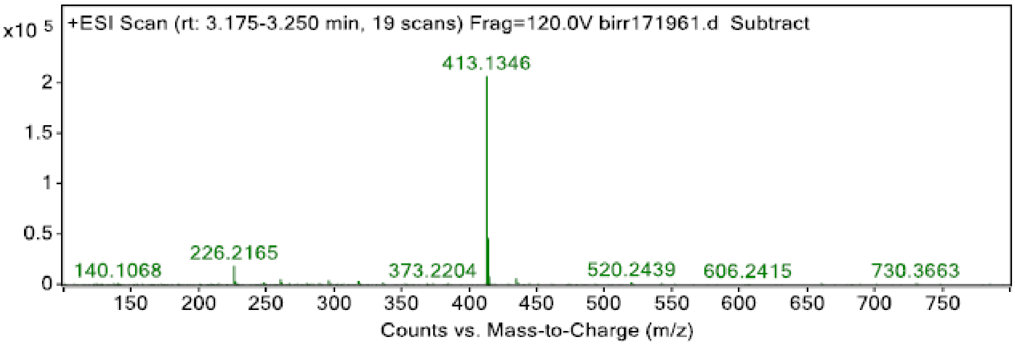

Figure S11: Mass spectrum of 7-(diethylamino)-2-oxo-2H-chromen-4-yl)methyl (4-nitrophenyl) carbonate$_{prot.}$.
MS (ESI) m/z calculated for $C_{21}H_{20}N_2O_7 + H^+$: 413.13 found: 413.1346.

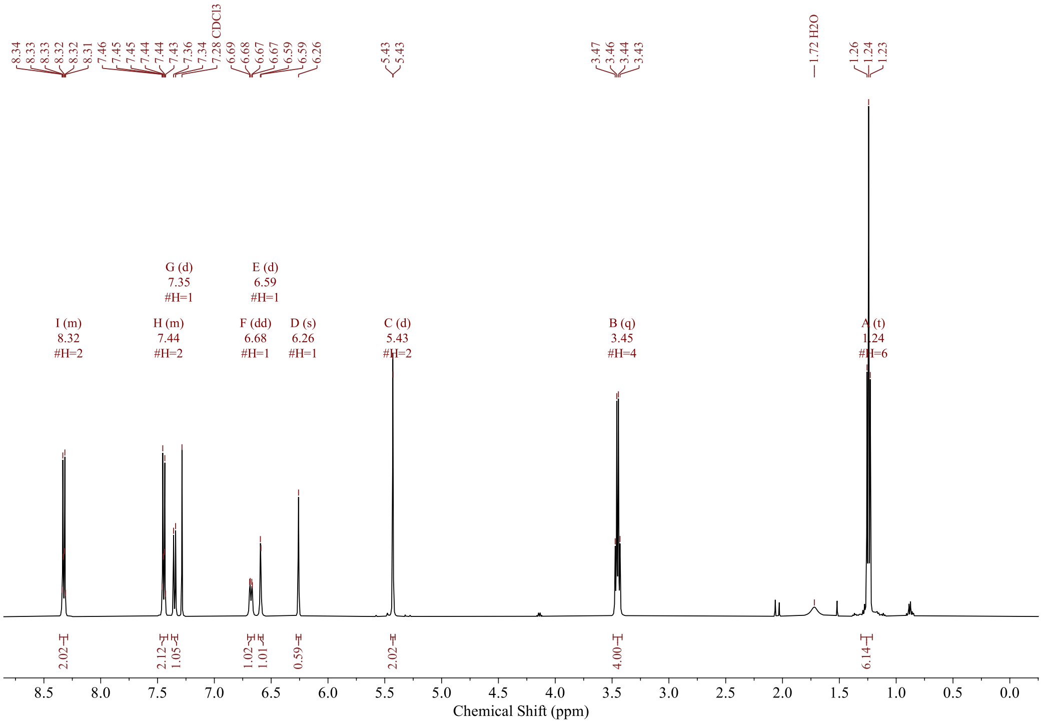

Figure S12: ^{1}H-NMR spectrum of 7-(diethylamino)-2-oxo-2H-chromen-4-yl)methyl (4-nitrophenyl) carbonate. ^{1}H-NMR (500 MHz, $CDCl_3$) δ(ppm) 8.36 – 8.29 (m, 2H), 7.48 – 7.41 (m, 2H), 7.35 (d, J = 9.0 Hz, 1H), 6.68 (dd, J = 9.3, 2.5 Hz, 1H), 6.59 (d, J = 2.6 Hz, 1H), 6.26 (s, 1H), 5.43 (d, J = 1.3 Hz, 2H), 3.45 (q, J = 7.1 Hz, 4H), 1.24 (t, J = 7.1 Hz, 6H).

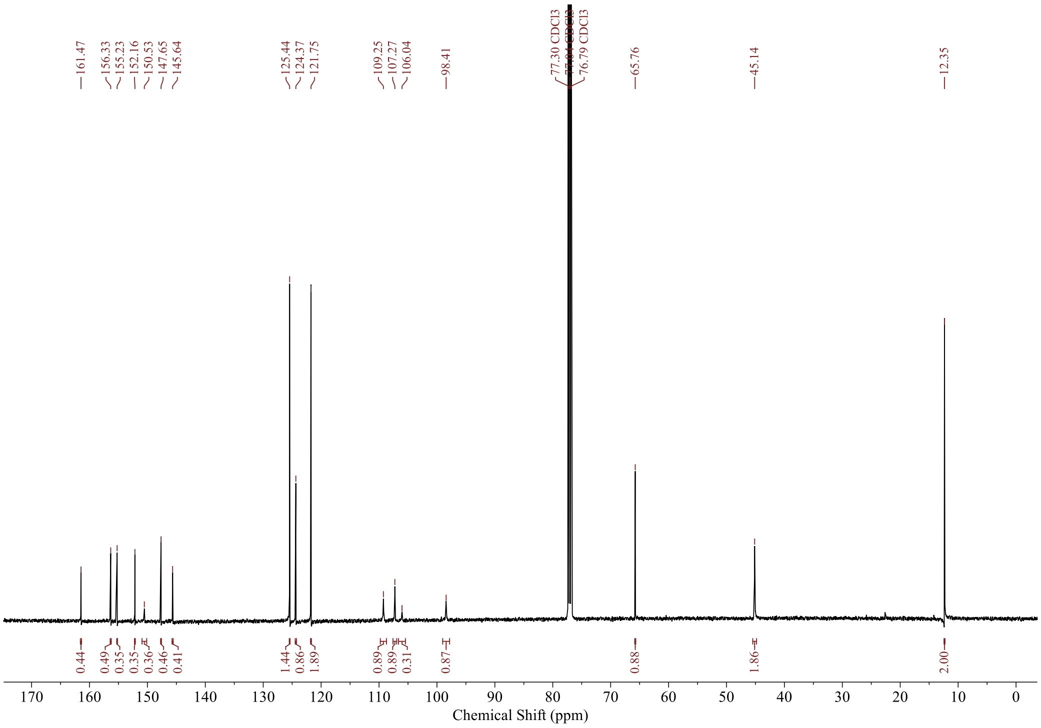

Figure S13: ^{13}C-NMR spectrum of 7-(diethylamino)-2-oxo-2H-chromen-4-yl)methyl (4-nitrophenyl) carbonate. ^{13}C-NMR (126 MHz, $CDCl_3$) δ(ppm) 161.47, 156.33, 155.23, 152.16, 150.53, 147.65, 145.64, 125.44, 124.37, 121.75, 109.25, 107.27, 106.04, 98.41, 65.76, 45.14, 12.35.

**Time [min]**	H_2O + 0.05 %TFA	ACN + 0.05 %TFA
0	95	5
1	95	5
16	5	95
20	5	95
20.3	95	5
25	95	5

Table S3: HPLC method for analysis of 7-(diethylamino)-2-oxo-2H-chromen-4-yl)methyl (4-nitrophenyl) carbonate. Flow rate: 1 mL

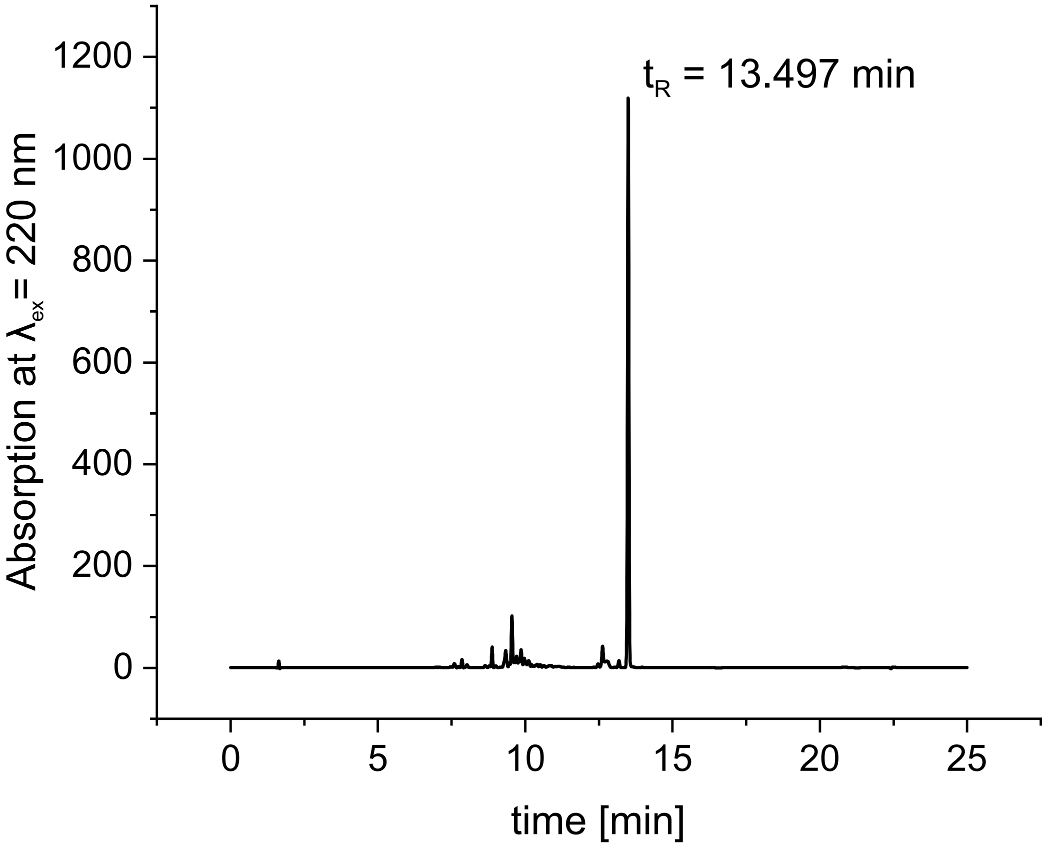

Figure S14: RP-HPLC analysis of 7-(diethylamino)-2-oxo-2H-chromen-4-yl)methyl (4-nitrophenyl) carbonate. Retention time t_R : 13.497 min. Purity of 69.84 %.

1.5 TAT 48-57 coupling to polymer

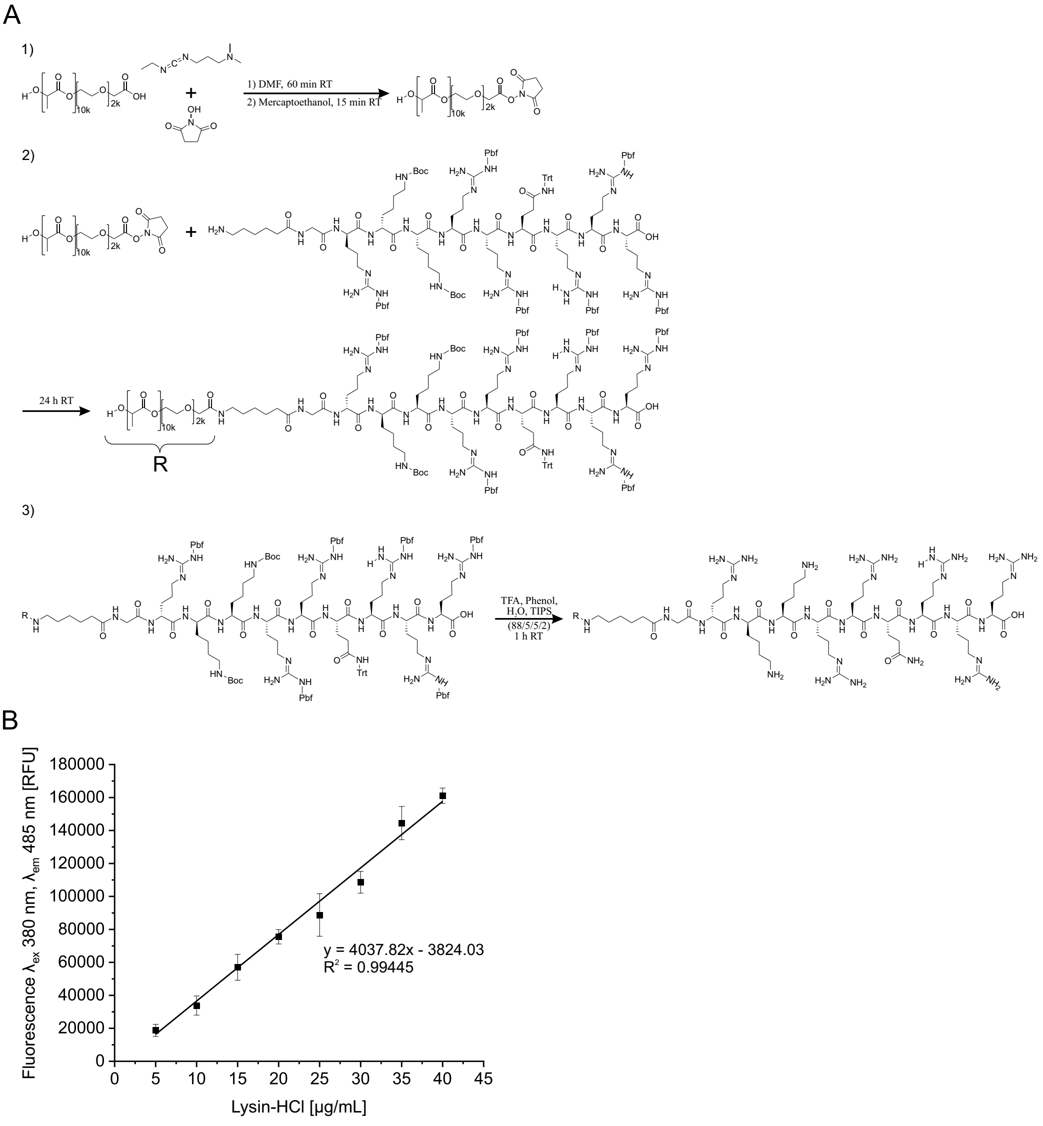

Figure S15: Functionalization of block copolymer and coupling efficiency. **A** 1) and 2) Reaction equation for the coupling of TAT 48-57 to the polymer via EDC/NHS chemistry. 3) Chemical equation for deprotection of sidechain protected TAT 48-57. **B** Plotting a calibration line for proofing linearity of Fluram assay by measuring the fluorescence of defined concentration of lysine hydrochloride after the addition of fluorescamine.

Calculation of lysine hydrochloride amount, which corresponds to 100 % coupling efficiency (CE).

$$m_{TAT-Polymer} = \frac{conc_{micelles}}{V_{sample}} \tag{3}$$

$$n_{Lysine\ in\ TAT\ 48-57} = \frac{m_{TAT-Polymer}}{M_{TAT-Polymer}} * 2 \tag{4}$$

$M_{COOH-PEG_{2k}-PLA_{10k}}$ determined by ^{1}H-NMR and $M_{TAT\ 48-57}$ was used to calculate $M_{TAT-Polymer}$.[1]

$$M_{TAT-Polymer} = M_{COOH-PEG_{2k}-PLA_{10k}} + M_{TAT48-57} \tag{5}$$

$$m_{Lysine-HCL(100\,\%)} = n_{Lysine\ in\ TAT\ 48-57} * M_{Lysine-HCl} \tag{6}$$

CE of TAT-Polymer was calculated with the following equation:

$$CE_{TAT-Polymer}[\%] = \frac{RFU_{TAT-Polymer}}{RFU_{Lysine-HCl\ (100\,\%)}} * 100 \tag{7}$$

The CE of TAT-Polymer reaction was 93.68 % ± 3.79 % (N=3).

1.6 Quantification of DEAC coupling to TAT-Polymer

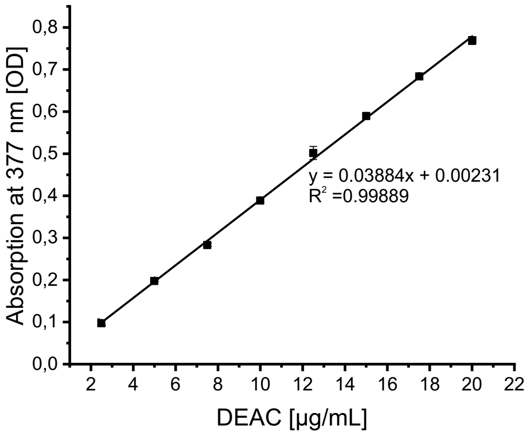

Figure S16: Coupling efficiency of DEAC to TAT-polymer. Plotting a calibration line for quantification of DEAC coupling to TAT-Polymer. Absorption is plotted against DEAC.

Assuming a complete reaction, two DEAC molecules couple to TAT-Polymer. The efficiency is calculated:

$$c_{\mathrm{DEAC}} = \frac{\beta_{\mathrm{DEAC}}}{\mathrm{M}_{\mathrm{DEAC}}} \tag{8}$$

$$c_{\mathrm{DEAC-TAT-Polymer}} = \frac{\beta_{\mathrm{DEAC-TAT-Polymer}}}{\mathrm{M}_{\mathrm{DEAC-TAT-Polymer}}} \tag{9}$$

$\mathrm{M}_{\mathrm{DEAC-TAT-Polymer}}$ was calculated as followed:

$$\mathrm{M}_{\mathrm{DEAC-TAT-Polymer}} = \mathrm{M}_{\mathrm{TAT-Polymer}} + (2 * \mathrm{M}_{\mathrm{DEAC}} - 2) \tag{10}$$

$$\mathrm{CE}_{\mathrm{DEAC-TAT-Polymer}}[\%] = \frac{\frac{c_{\mathrm{DEAC}}}{c_{\mathrm{DEAC-TAT-Polymer}}}}{2} * 100 \tag{11}$$

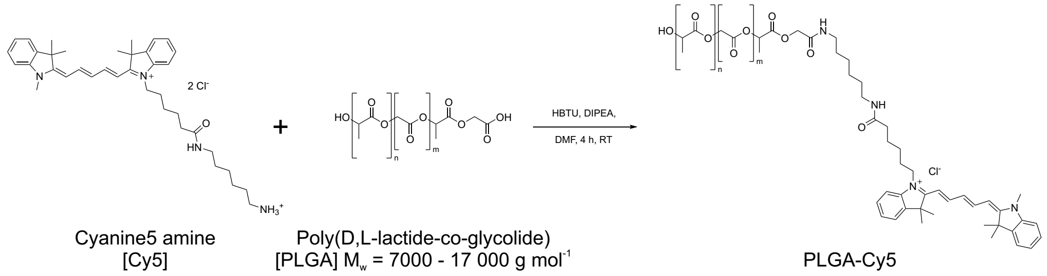

Scheme S5: Chemical equation for synthesis of Cy5 coupling to PLGA.

2 Calculation on Foerster radius and characterization of PEG conformation

The Foerster Radius (R_0) was calculated with Equation (12)

$$R_0 = 0.211 * \sqrt[6]{\kappa^2 n^{-4} Q_D J(\lambda)} \tag{12}$$

where κ^2 is the orientation factor, n is the refractive index, Q_D is the quantum yield of the donor and $J(\lambda)$ is the overlap integral calculated separately by Equation (13). [2]

$$J(\lambda) = \frac{\int_0^\infty F_D(\lambda)\varepsilon_A(\lambda)\lambda^4\, d\lambda}{\int_0^\infty F_D(\lambda)\, d\lambda} \tag{13}$$

$\varepsilon_A(\lambda)$ is the extinction coefficient of the acceptor fluorophore at a defined wavelength, F_D is the fluorescence spectrum of the donor. For the calculation of Foerster radius for the FRET pair DEAC-Cy5, κ^2 was assumed 2/3 and Q_D was obtained from literature with a value of 0.079.[3] R_0 was calculated to be 32.6 Å or 3.26 nm respectively.

The PEG chains in core-shell NP design can arrange either as a mushroom or a brush conformation. The conformation is described by the quotients of Flory Radius (R_F) and grafting distance (D) of PEG chains which was previously described by our group and others.[1, 4] A value of $R_F/N < 1$ is linked to mushroom conformation and $R_F/N > 1$ is referred as brush conformation, which can be further characterized as dense brush with a $R_F/N > 2.8$.[4] R_F is defined by Equation (14), where α is the length of a PEG monomer (3.5 nm) and N is the number of monomers in PEG chain calculated by dividing the molecular weight of the PEG chain (2000 g mol^{-1}) by the monomer molecular weight (44 g mol^{-1}).

$$R_F = \alpha * N^{\frac{3}{5}} \tag{14}$$

For the PEG_{2k}-PLA_{10k} block copolymer used in our NP design R_F has a value of 3.46 nm. The grafting distance (D) between two polymer chains on a particle surface is calculated by Equation (15), where S corresponds to the surface occupied by a single PEG chain.[5]

$$D = 2 * \sqrt[2]{\frac{S}{\pi}} \tag{15}$$

S is given by:

$$S = \frac{6 * M_{PEG}}{d_h * N_A * f * \rho_{NP}} \tag{16}$$

where, M_{PEG} is the molecular weight of the PEG chain, d_h is the hydrodynamic diameter of the NP, N_A is the Avogadro number, f is the mass fraction of PEG in the blends of the PEG_{2k}-PLA_{10k} block copolymer and ρ_{NP} is the density of the NP. The density of 1.25 g^2 cm^{-1} for NP was estimated corresponding to the density of NP composed of PLA-g-PEG blends.[6] D was calculated to be 0.99 nm, resulting in a value for $R_F/N = 3.58$. The thickness of the PEG layer was calculated by Equation (17) had a value of 8.28 nm.[4]

$$L = \frac{N * \alpha^{5/3}}{D^{2/3}} \tag{17}$$

Thus, it could be assumed that the polymer chains arrange in a brush conformation.

3 Photobleaching

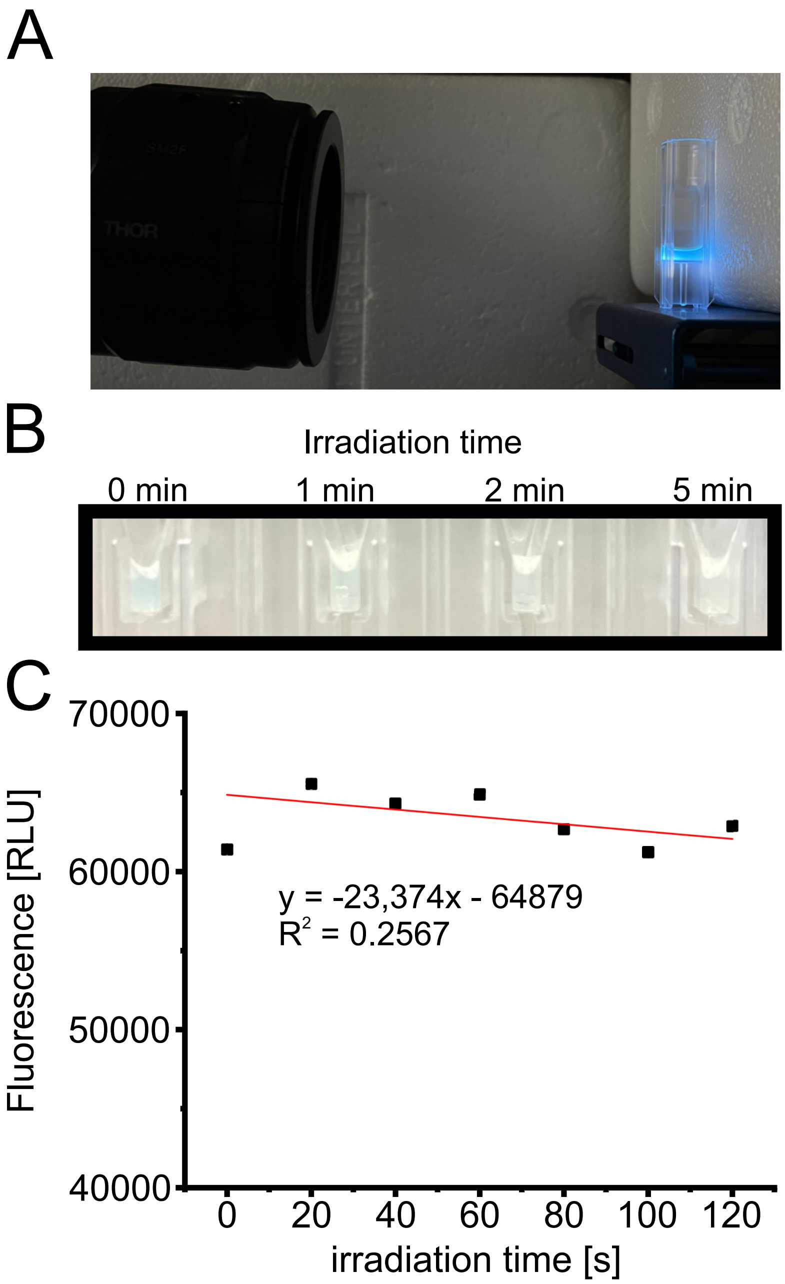

Figure S17: Photobleaching of NP. **A** Irradiation setup. **B** Image of [TAT]-NP before and after different irradiation times. **C** Fluorescence of Cy5 of COOH-NP is plotted against irradiation time of NP with LED at $\lambda = 365$ nm.

4 Nanoparticle characterization

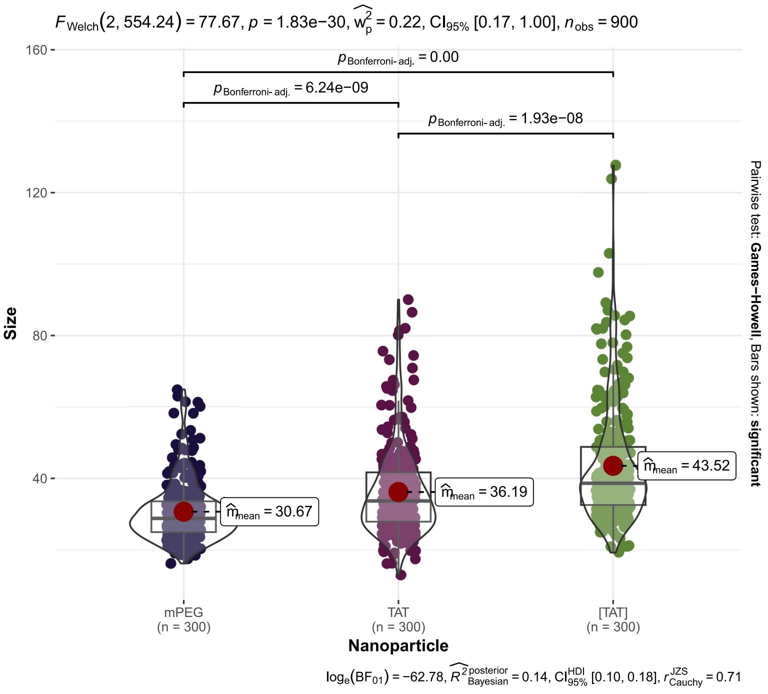

Figure S18: Size distribution and statistics of TEM dataset. Violin plots of NP size distributions depicting the full kernel density of measured sizes. The median NP size is shown as horizontal line within the box, whose lower and upper edges represent the first (Q1) and third (Q3) quartiles, respectively. Whiskers indicate the most extreme data points within 1.5 times the interquartile range from the lower and upper quartiles. Red dots indicate mean sizes. A one-way Welch's ANOVA with p-values < 0.001 (adjusted using Bonferroni method) showed significant differences in NP mean size.

References

[1] Kathrin Schorr et al. "The quantity of ligand-receptor interactions between nanoparticles and target cells". In: *Nanoscale horizons* 10.4 (2025), pp. 803–823. DOI: `10.1039/D4NH00645C`.

[2] P. Wu and L. Brand. "Resonance energy transfer: methods and applications". In: *Analytical biochemistry* 218.1 (1994), pp. 1–13. ISSN: 0003-2697. DOI: `10.1006/abio.1994.1134`.

[3] André Vidal Pinheiro et al. "pH effect on the photochemistry of 4-methylcoumarin phosphate esters: caged-phosphate case study". In: *The journal of physical chemistry. A* 114.49 (2010), pp. 12795–12803. DOI: 10.1021/jp103045u.

[4] Qi Yang and Samuel K. Lai. "Engineering Well-Characterized PEG-Coated Nanoparticles for Elucidating Biological Barriers to Drug Delivery". In: *Methods in molecular biology (Clifton, N.J.)* 1530 (2017), pp. 125–137. DOI: 10.1007/978-1-4939-6646-2{\textunderscore}8.

[5] R. Gref et al. "'Stealth' corona-core nanoparticles surface modified by polyethylene glycol (PEG): influences of the corona (PEG chain length and surface density) and of the core composition on phagocytic uptake and plasma protein adsorption". In: *Colloids and Surfaces B: Biointerfaces* 18.3-4 (2000), pp. 301–313. ISSN: 09277765. DOI: 10.1016/S0927-7765(99)00156-3.

[6] Jean-Michel Rabanel et al. "Effect of the Polymer Architecture on the Structural and Biophysical Properties of PEG-PLA Nanoparticles". In: *ACS applied materials & interfaces* 7.19 (2015), pp. 10374–10385. DOI: 10.1021/acsami.5b01423.
